# Targeted biologic inhibition of both tumor cell-intrinsic and intercellular CLPTM1L/CRR9-mediated chemotherapeutic drug resistance

**DOI:** 10.1038/s41698-021-00152-9

**Published:** 2021-03-02

**Authors:** Deepak Parashar, Anjali Geethadevi, Donna McAllister, Johnathan Ebben, Francis C. Peterson, Davin R. Jensen, Erin Bishop, Sunila Pradeep, Brian F. Volkman, Michael B. Dwinell, Pradeep Chaluvally-Raghavan, Michael A. James

**Affiliations:** 1 Department of Obstetrics and Gynecology, Medical College of Wisconsin, Milwaukee, WI USA; 2 Department of Microbiology and Immunology, Medical College of Wisconsin, Milwaukee, WI USA; 3 Department of Medicine, University of Wisconsin, Madison, WI USA; 4 Department of Biochemistry, University of Wisconsin, Madison, WI USA; 5 Essential Biotechnology LLC, Big Bend, WI United States

**Keywords:** Cancer therapeutic resistance, Ovarian cancer, Pancreatic cancer, Non-small-cell lung cancer, Drug development

## Abstract

Recurrence of therapy-resistant tumors is a principal problem in solid tumor oncology, particularly in ovarian cancer. Despite common complete responses to first line, platinum-based therapies, most women with ovarian cancer recur, and eventually, nearly all with recurrent disease develop platinum resistance. Likewise, both intrinsic and acquired resistance contribute to the dismal prognosis of pancreatic cancer. Our previous work and that of others has established CLPTM1L (cleft lip and palate transmembrane protein 1-like)/CRR9 (cisplatin resistance related protein 9) as a cytoprotective oncofetal protein that is present on the tumor cell surface. We show that CLPTM1L is broadly overexpressed and accumulated on the plasma membrane of ovarian tumor cells, while weakly or not expressed in normal tissues. High expression of CLPTM1L is associated with poor outcome in ovarian serous adenocarcinoma. Robust re-sensitization of resistant ovarian cancer cells to platinum-based therapy was achieved using human monoclonal biologics inhibiting CLPTM1L in both orthotopic isografts and patient-derived cisplatin resistant xenograft models. Furthermore, we demonstrate that in addition to cell-autonomous cytoprotection by CLPTM1L, extracellular CLPTM1L confers resistance to chemotherapeutic killing in an ectodomain-dependent fashion, and that this intercellular resistance mechanism is inhibited by anti-CLPTM1L biologics. Specifically, exosomal CLPTM1L from cisplatin-resistant ovarian carcinoma cell lines conferred resistance to cisplatin in drug-sensitive parental cell lines. CLPTM1L is present in extracellular vesicle fractions of tumor culture supernatants and in patients’ serum with increasing abundance upon chemotherapy treatment. These findings have encouraging implications for the use of anti-CLPTM1L targeted biologics in the treatment of therapy-resistant tumors.

## Introduction

Nine of ten ovarian cancer patients succumb to recurrent, chemoresistant disease, presenting a dire unmet need. Resistance to therapy remains an obstacle to successful treatment of most cancer. For instance, in pancreatic cancer particularly, a challenging tumor microenvironment and unknown factors contribute to a strong resistance to therapy. Processes intrinsic to tumor cells as well as those stemming from the tumor milieu can promote a cellular stress response, EMT, quiescence, stem phenotypes, and autophagy, contributing to drug and immune resistance^1–5^. Factors outside the tumor cell compartment that can confer resistance include exosomal or paracrine factors secreted from either stromal cells or tumor cells themselves^6–9^.

We have recently identified surface-expressed CLPTM1L to be a tumor-specific cytoprotective and chemoresistance factor in pancreatic and other tumor types that can be further induced by genotoxic or endoplasmic reticular stress^2,10–12^. CLPTM1L has recently been found to be critical for embryonic and neonatal survival but dispensable in adult animals^13^. Trezise et al. also found CLPTM1L to be highly and selectively expressed on the surface of multiple myeloma cells^13^. Several other studies have demonstrated robust overexpression of CLPTM1L on the surface of malignant cells in a variety of tumor types with minimal non-surface expression or no expression in normal tissues^2,10–12,14–17^. In the current study, we describe overexpression of CLPTM1L in greater than 90% of ovarian squamous carcinomas tested and an association of higher expression with poor survival. With survival rates in ovarian carcinoma depending largely on sustained response to platinum based therapies, primarily carboplatin, we sought to determine if CLPTM1L contributes to platinum resistance in a therapeutically targetable manner.

Herein we demonstrate re-sensitization of chemotherapy resistant human ovarian tumor cells to carboplatin and pancreatic tumor cells to gemcitabine by human anti-CLPTM1L mAbs as first-in-class biologic drug candidates. We have previously shown that inhibition of CLPTM1L can chemosensitize pancreatic ductal adenocarcinoma to gemcitabine treatment^10–12^. In the present study, CLPTM1L protein was found to be more abundant in platinum pre-treated ovarian tumor cells, and anti-CLPTM1L treatment was synergistic with carboplatin killing. Treatment of C57bl/6 mice in a syngeneic model of disseminated peritoneal ovarian cancer with human anti-CLPTM1L inhibited tumorigenesis and potentiated the therapeutic activity of carboplatin. Likewise, anti-CLPTM1L treatment restored platinum sensitivity to novel patient-derived parental and cisplatin-conditioned ovarian tumor cell and spheroid cultures, both in vitro and in vivo.

We present the novel finding that in addition to cell-autonomous effects of CLPTM1L on chemoresistance, CLPTM1L in cell culture supernatants and extracellular vesicle fractions can confer intercellular chemoresistance to bystander ovarian and pancreatic tumor cells. Extracellular vesicle CLPTM1L abundance was increased upon pre-treatment with chemotherapy.

With relatively long remissions and favorable co-morbidity profiles after first line therapy, ovarian carcinoma is particularly amenable to early clinical trials and maintenance therapy with targeted biologics. Tumoricidal activity and improvement of therapeutic index by inhibition of a novel mechanism of both cell-intrinsic and intercellular (extracellular vesicle) cytoprotection encourages further investigation of anti-CLPTM1L mAbs as oncology drug candidates.

## Results

### Over-expression of CLPTM1L in ovarian serous adenocarcinoma and association with poor outcome

**Fig. 1:**
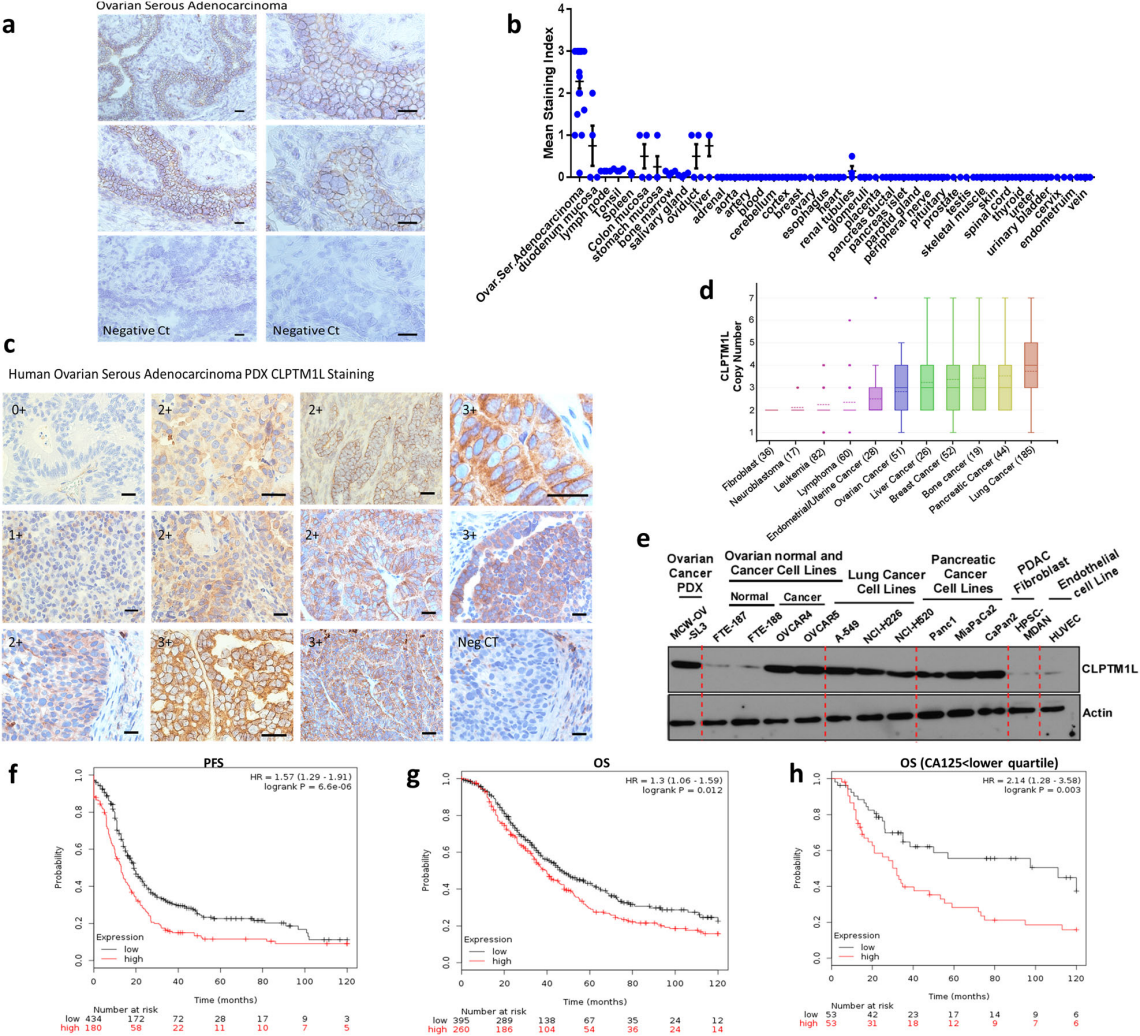
CLPTM1L expression and outcome. **a** CLPTM1L IHC on a representative human ovarian serous adenocarcinoma. Negative CT: secondary antibody staining only. Bars = 50 µM. **b** IHC staining indices for ovarian serous adenocarcinoma (*n* = 24) (represented in ‘**a**’) and FNA normal human tissues *n* = 144 (*n* = 4 per tissue). Bars represent means, and error bars represent standard error of the mean. **c** CLPTM1L staining on each of 11 patient-derived xenograft tissues. Negative control in the lower right panel was performed on the same patient and section as in the lower left panel. Scale bars = 50 µM. **d** Copy number variation (CNV) of CLPTM1L in various cancer cell lines analyzed using the depmap portal (https://depmap.org/portal). Box limits represent upper and lower quartiles. Whiskers represent 1.5× inter-quartile range. Points represent outliers. **e** Western blotting for CLPTM1L in human ovarian cancer PDX, endothelial, normal fallopian tube epithelial cells, pancreatic fibroblasts, and different tumor cell lines. **f** Progression free survival. **g** Overall survival. **h** Overall survival in patients with CA125 < lower quartile as analyzed using KM Plot.

To determine the level of CLPTM1L protein expression in ovarian serous adenocarcinoma, we conducted immunohistochemistry on frozen histology sections of treatment-naïve tumors from 24 patients. The majority (87.5%) of tumors exhibited a moderate to strong staining intensity index (1.5-3 on a 0-3 scale) with an apparent staining of the plasma membrane of tumor cells (Fig. 1a). The overall average staining index was 2.28. In contrast, 144 normal tissues on an FDA tissue microarray (*n* = 4 per tissue) demonstrated weak to no staining, with an average index of 0.089 (*T*-test *p* < 2 × 10^−5^) (Fig. 1b). Ten of eleven (91%) naïve and platinum-treated human ovarian serous adenocarcinoma patient-derived xenograft tissues stained positively for CLPTM1L with an average staining index of 1.82 (Fig. 1c). Patient-derived xenograft data was not included in quantitative analysis and comparison to normal tissues as in Fig. 1b because it is not directly comparable to IHC on human tissues. There was no statistical difference in expression between naïve and treated tumors with resolution of differences requiring larger samples sizes. Expression was predominantly in tumor tissues. Some immune (plasma) cells appeared to express CLPTM1L but stromal fibroblasts and endothelial cells were negative by IHC (Fig. 1a, c). Moreover, we investigated copy number variation (CNV) of various cancer cell lines using the depmap portal (https://depmap.org/portal) and found that ovarian cancer (*n* = 51) is among several tumor cell lines with high CNV of CLPTM1L compared to fibroblasts (*n* = 36) (Fig. 1d). Further, we investigated CLPTM1L protein levels using western blotting of lysates from patient-derived ovarian cancer (MCW-OV-SL-3), normal fallopian tube epithelial (FTE187 and FTE 188), ovarian cancer (OVCAR4 and OVCAR5), lung cancer (A549, NCI-H226, and NCI-H520), pancreatic cancer (Panc1, MiaPaCa2, and CaPan2), immortalized activated human pancreatic stellate (HPSC)-fibroblast and endothelial (HUVEC) cell lines. We found that CLPTM1L was expressed highly in ovarian cancer, lung cancer and in pancreatic cancer cell lines but not in normal ovarian cell lines, fibroblasts or endothelial cell lines (Fig. 1e). Kaplan–Meier analysis of the effect of CLPTM1L expression on progression free survival in ovarian cancer patients with TCGA data using KM Plotter^18^ showed an association of high CLPTM1L expression with poor outcome with a hazard ratio of 1.57 (log rank *p* = 6.6 × 10^−6^) (Fig. 1f). High CLPTM1L expression was also associated with poor overall survival with a hazard ratio of 1.3 (*p* = 0.012) (Fig. 1g), particularly for those patients with a CA125 tumor marker level below the lowest quartile (HR = 2.14, *p* = 0.003) (Fig. 1h). The significance of increased association of CLPTM1L expression with outcome in this population is not yet clear.

### Development of human IgG1 anti-CLPTM1L monoclonal antibody for re-sensitization of ovarian tumor cells to platinum-based drugs and anti-tumorigenesis

Given our previous demonstration of chemosensitization of pancreatic tumor cells to chemotherapeutic killing and inhibition of anchorage independent growth and tumorigenesis using murine monoclonal antibodies (mAbs) targeting CLPTM1L^12^, we developed fully human antibodies with antagonism for CLPTM1L.

Given superior chemosensitization and cytotoxic efficacy of mAbs raised against the “lead” epitope as described in ref. ^12^, we used peptide antigen representing this epitope to screen a naïve human scFv (single chain variable fragment) phage library for human CLPTM1L-inhibitory antibodies. Five scFv clones specifically binding our epitope resulted from the screen. These antibodies were expressed transiently in 293T cells as both scFv fragments and as codon-optimized scFv-Fc (IgG1) fusions and purified. Codon optimized variable sequences of clone 102-5 was expressed transiently in Chinese hamster ovary cells as full-length human IgG1. Full characterization of 102-5 demonstrating affinity and specificity to CLPTM1L is provided in Supplementary Fig. 1a–i.

**Fig. 2:**
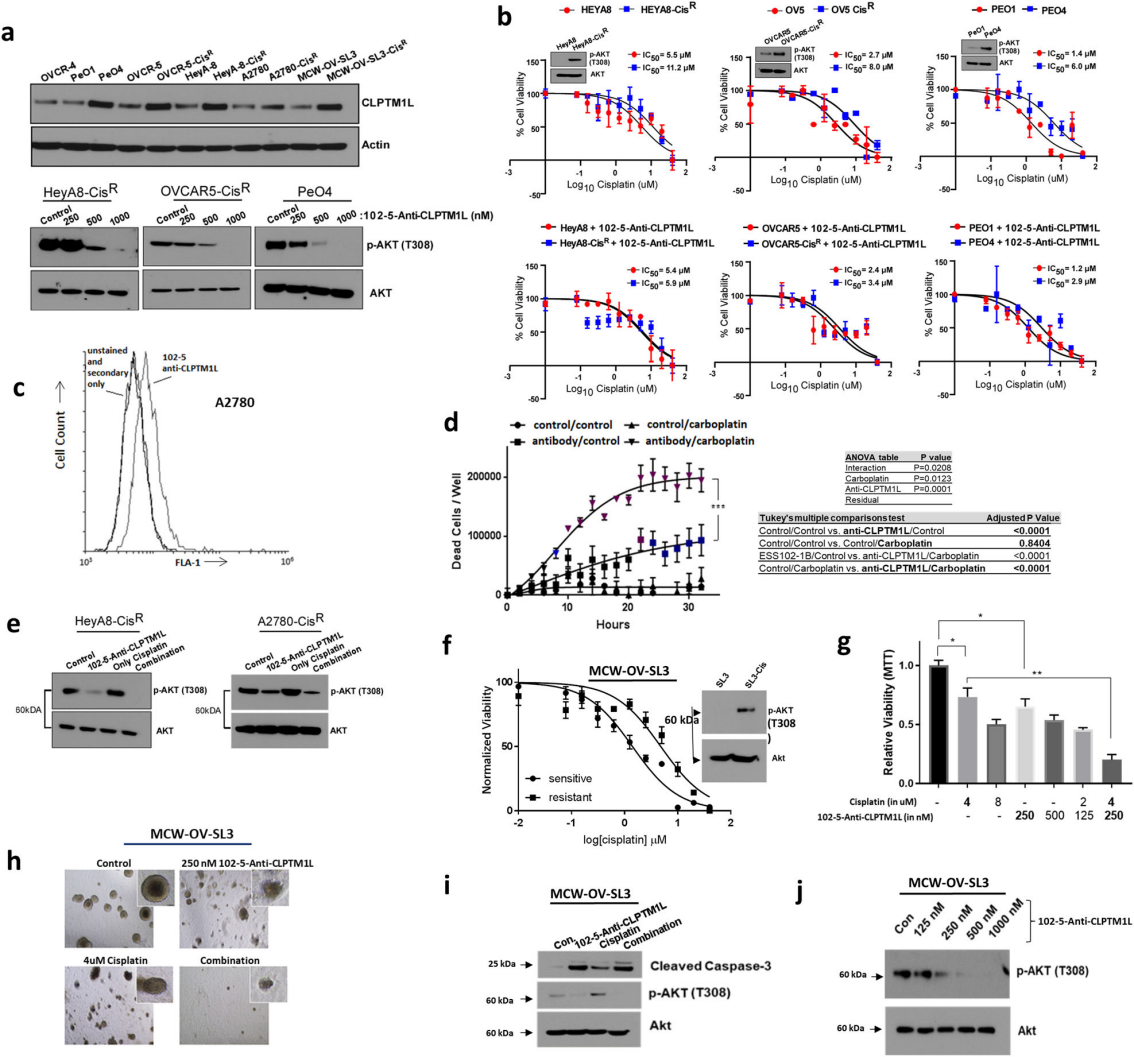
Resensitization to chemotherapeutic killing by 102-5 anti-CLPTM1L. **a** Western blotting of cisplatin-sensitive and resistant ovarian tumor cell lysates for CLPTM1L and analysis of dose–response of phospho-Akt and total Akt to 102-5 anti-CLPTM1L treatment. **b** Dose–response analyses and cisplatin IC50s of cisplatin-sensitive and resistant human ovarian tumor cells. Error bars represent standard error of the mean. Akt phosphorylation levels in resistant versus resistant lines are shown in insets. **c** Flow cytometry on A2780 detecting cell surface CLPTM1L with 102-5 human anti-CLPTM1L. **d** Kinetic killing assay (live imaging) in A2780 with human anti-CLPTM1L and/or 10 µM carboplatin treatment. Error bars represent standard error of the mean. *p < 0.05, **p < 0.005, ***p < 0.0005. **e** Western blotting for Akt and phospho-Akt (T308) in A2780 and HeyA8 resistant cell lines treated with 102-5 anti-CLPTM1L and/or cisplatin. **f** Cisplatin dose–response curves for MCW-OV-SL3 sensitive (parental) and resistant (MCW-OV-SL3-Cis^R^) tumor spheroids, pAkt (T308) and total Akt western blots on MCW-OV-SL3 and MCW-OV-SL3-Cis^R^ spheroid lysates (inset). Error bars represent standard error of the mean. **g** Viability (MTT) of MCW-OV-SL3-Cis^R^ spheroids after 16 days of growth, treated with cisplatin and/or 102-5 anti-CLPTM1L at the indicated concentrations beginning at 4 days post-seeding. *p < 0.05, **p < 0.005. **h** Photomicrographs of MCW-OV-SL3-Cis^R^ spheroids after 16 days of growth, treated with cisplatin and/or 102-5 anti-CLPTM1L. **i** Western blotting of MCW-OV-SL3-Cis^R^ spheroid lysates for caspase cleavage and Akt phosphorylation after 16 days of growth, treated with cisplatin and/or 102-5 anti-CLPTM1L. **j** Western blotting of MCW-OV-SL3-Cis^R^ monolayer cultures for phospho- and total Akt following treatment with control (PSB) or 102-5 anti-CLPTM1L at the indicated concentrations.

Next, we evaluated the levels of CLPTM1L and cancer stem cell markers in a panel of high-grade serous ovarian carcinoma (HGSOC), ovarian cystadenocarcinoma, and endometrioid ovarian cancer cell lines and found that CLPTM1L is expressed highly in cisplatin resistant ovarian tumor cells PeO4, OVCAR5-Cis^R^, HeyA8-Cis^R^, A2780-Cis^R^, and MCW-OV-SL3-Cis^R^ compared to their parent cells (Fig. 2a) along with increased expression of c-KIT, and CD44 and a reduction in CD24, which are features of ovarian cancer stem cells (Supplementary Fig. 2a). Next, we used the MCW-OV-SL3 cell line, which is a patient-derived endometrioid ovarian cancer subtype cell line, and its cisplatin-resistant derivative, which was generated through chronic exposure to cisplatin (MCW-OV-SL3-Cis^R^) and other cisplatin-resistant cell lines to determine the expression of cancer stem cell markers and their spheroid formation abilities upon treatment with 102-5 anti-CLPTM1L. We found that treatment with 102-5 anti-CLPTM1L resulted in a single major band consistent with 62 kDa CLPTM1L, which concurred with results using commercial anti-CLPTM1L and demonstrated upregulation of CLPTM1L accumulation in cisplatin-resistant ovarian tumor cells compared to parental cisplatin-sensitive cells (Fig. 2a and Supplementary Figs. 1b and 2). We also observed that the treatment of cisplatin resistant HeyA8-Cis^R^, OVCAR5-Cis^R^, PeO4 cells with 102-5 anti-CLPTM1L decreased Akt phosphorylation at threonine 308 (T308) in a dose-dependent manner (Fig. 2a).

Cisplatin dose–response curves were generated for HGSOC and ovarian cystadenocarcinoma cells and their cisplatin-resistant derivatives treated with 102-5 anti-CLPTM1L or isotype control antibody (Fig. 2b). IC50 values for cisplatin were calculated for each line and for those treated with antibodies. Treatment with 102-5 anti-CLPTM1L significantly lowered cisplatin IC50s in HeyA8-Cis^R^ (11.2 µM, 95% confidence interval [CI] 8.3–15.8 vs. 5.9 µM [CI] 3.7–9.3, *p* = 0.0056), OVCAR5-Cis^R^ (8.8 µM [CI] 6.6–11.85 vs. 3.4 [CI] 2.2–5.7, *p* = 5.5 × 10^−5^) and PeO4 (6.0 µM [CI] 4.2–8.6 vs. 2.9 [CI] 1.7–4.9, *p* = 4.2 × 10^−5^) cisplatin-resistant HGSOC and ovarian cystadenocarcinoma cell lines (Fig. 2b). Treatment of these resistant cell lines with 102-5 anti-CLPTM1L restored cisplatin sensitivity to statistical equivalence with their respective cisplatin-sensitive parental cells lines (*p* = 0.78, 0.43, and 0.06 for HeyA8-Cis^R^, OVCAR5-Cis^R^, and PeO4, respectively).

Using flow cytometry, gated as shown in Supplementary Fig. 3, binding of full-length human anti-CLPTM1L (102-5) to the surface of live human ovarian tumor cells (A2780) was demonstrated (Fig. 2c). On-cell western analysis also demonstrated detection of cell-surface CLPTM1L on live A2780 and MCW-OV-SL3-Cis^R^ cells (Supplementary Fig. 1e).

Live cell imaging measurement of cell death over a time course of 30 h demonstrated killing by human anti-CLPTM1L (102-5) alone but not by 10 µM carboplatin and potentiation of killing by carboplatin in combination with 102-5 anti-CLPTM1L (Fig. 2d). The combination of 102-5 anti-CLPTM1L and carboplatin resulted in robust killing with a highly significant interaction between anti-CLPTM1L treatment and carboplatin treatment (ANOVA *p* < 0.02). 102-5 anti-CLPTM1L treatment resulted in decreased Akt phosphorylation at threonine 308 in both HeyA8-Cis^R^ and A2780-Cis^R^ cells, including those treated in combination with cisplatin (Fig. 2e). Similarly, OVCAR4 human ovarian serous adenocarcinoma cells were sensitized to carboplatin by treatment with 102-5 anti-CLPTM1L (Supplementary Fig. 4a). MCW-OV-SL3 primary, patient-derived ovarian endometrioid carcinoma cells were treated with cisplatin to generate a cisplatin resistant cell line (Fig. 2f). Importantly, in concordance with the data that induction of CLPTM1L in MCW-OV-SL3-cisplatin resistant cells in Fig. 2c, phosphorylation of Akt at threonine 308 was increased in MCW-OV-SL3-Cis^R^ compared to its parental cells (Fig. 2f). Treatment of cisplatin resistant MCW-OV-SL3-Cis^R^ cells with 102-5 anti-CLPTM1L abrogated resistance to cisplatin in a dose-dependent manner as measured by spheroid viability, size, and number over 16 days in culture with cisplatin and anti-CLPTM1L treatment beginning at day 4 in culture (Fig. 2g and h and Supplementary Fig. 4c). While anti-CLPTM1L treatment alone was sufficient to inhibit anchorage independent growth of MCW-OV-SL3-Cis^R^ and parental MCW-OV-SL3 cells in a dose-dependent manner (Supplementary Fig. 4b)(ANOVA *p* < 0.0005), re-sensitization of MCW-OV-SL3-Cis^R^ cells to cisplatin killing by 102-5 anti-CLPTM1L was synergistic as measured by two-way ANOVA (*p* < 0.05) and isobole combination index (0.65)(Chou-Talalay methodology)(Fig. 2g and h and Supplementary Fig. 4c and d). Spheroid growth was also inhibited in a dose-dependent manner by 102-5 anti-CLPTM1L in cisplatin-resistant, stem-like cell lines HeyA8-Cis^R^, OVCAR5-Cis^R^ (HGSOC), and PeO4 (ovarian cystadenocarcinoma) (Supplementary Fig. 2). Caspase-3 cleavage was induced by both cisplatin treatment and 102-5 anti-CLPTM1L treatment, with signal being stronger in lysates from anti-CLPTM1L-treated MCW-OV-SL3-Cis^R^ spheroids compared to cisplatin-treated spheroids (Fig. 2i). Akt phosphorylation at threonine 308 was induced by cisplatin treatment but abrogated by 102-5 anti-CLPTM1L treatment in these spheroids (Fig. 2i). Furthermore, 102-5 anti-CLPTM1L treatment resulted in a dose-dependent inhibition of Akt phosphorylation at threonine 308 in monolayer cultures of MCW-OV-SL3-Cis^R^, HeyA8-Cis^R^, OVCAR5-Cis^R^ and PeO4 cells, (Fig. 2a and j). Moreover, 102-5 anti-CLPTM1L treatment also inhibit p-Akt (T308) and 3-D spheroid formation in lung cancer cell lines (A549, NCI-H520, NCI-H226) (Supplementary Fig. 5a–c) and in pancreatic cancer cell lines (Panc1, MiaPaca2, and Capan2), (Supplementary Fig. 5d–f).

### CLPTM1L ectodomain-dependent intercellular chemoresistance and its inhibition by CLPTM1L mAb

**Fig. 3:**
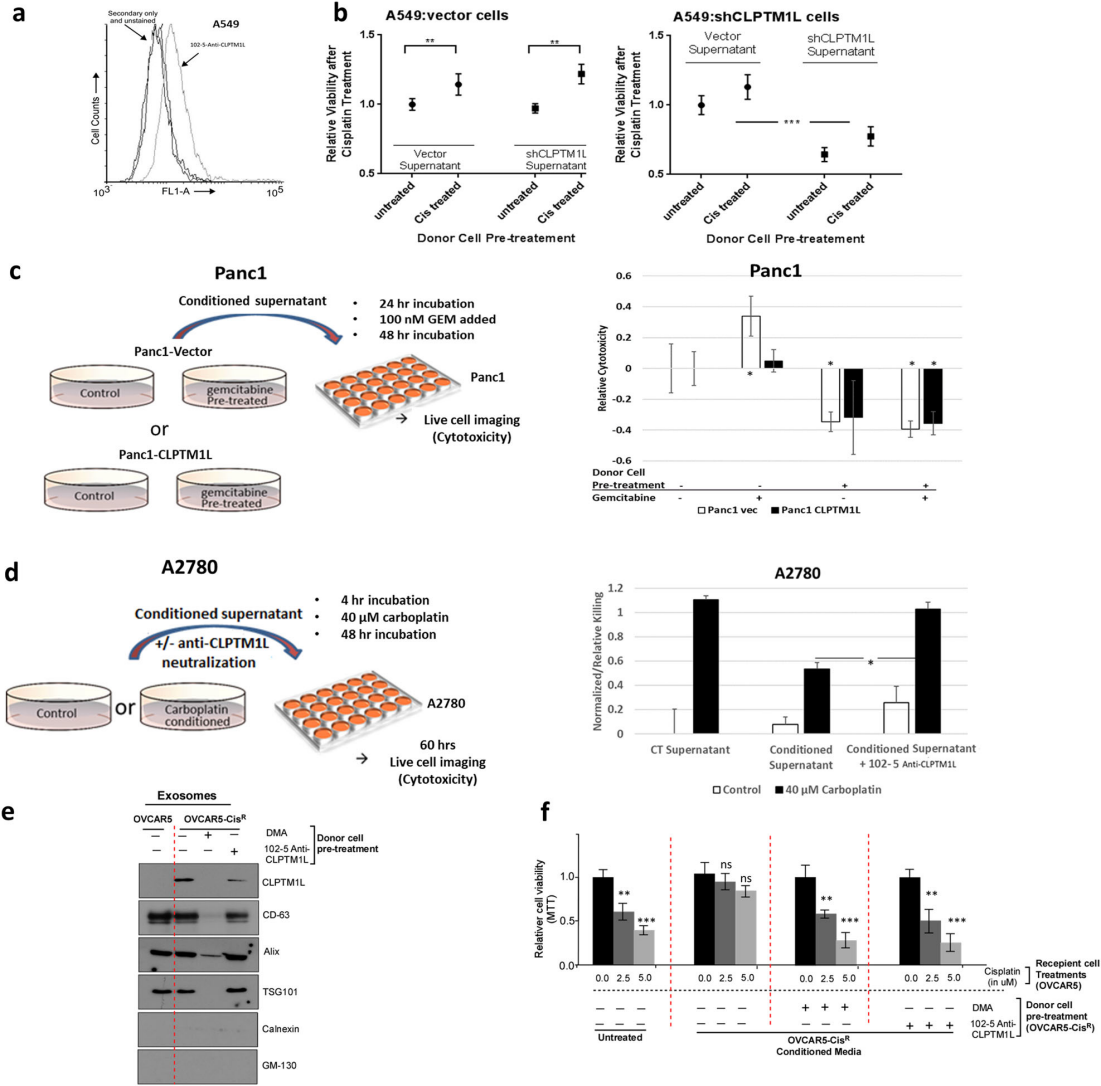
Chemoprotection by culture supernatants. **a** Flow cytometry histograms of fluorescent labeling of live A549 cells with 102-5 anti-CLPTM1L primary and Alexafluor-488-conjugated secondary antibodies. **b** Relative viability (MTS) of A549 cells (vector control, or with CLPTM1L shRNA knockdown (shCLPTM1L) after exposure to cisplatin and culture supernatants from either vector control or CLPTM1L shRNA cells that were untreated or pre-treated with cisplatin (*x* axis). Error bars represent standard error of the mean. **p = 0.003, ***p = 0.0001. **c** Relative killing (live imaging cytotoxicity) of Panc1 pancreatic tumor cells after 24-h treatment with 100 nM gemcitabine and 72-h culture supernatants that were either untreated or pre-treated with 20 nM gemcitabine. Error bars represent standard error of the mean. **d** Relative killing by 40 µM carboplatin (live imaging cytotoxicity) of ovarian tumor cells treated with supernatants from control or 10 µM carboplatin-conditioned A2780 donor cells at 60 h post-carboplatin treatment. Supernatants in the 102-5 groups were pre-treated with 100 nM 102-5 anti-CLPTM1L overnight. Error bars represent standard error of the mean. *p < 0.05, **p < 0.005. **e** OVCAR5–Cis^R^ cells were treated with or without DMA or 102-5 Anti-CLPTM1L and western blotting was performed from the exosomes isolated from the conditioned media using indicated antibodies. **f** Results of cell viability assays performed on OVCAR5 cells treated with or without cisplatin that were untreated or pre-treated with culture supernatants of OVCAR5-Cis^R^ cells with or without DMA or 102-5 Anti-CLPTM1L (*x* axis). Error bars represent standard error of the mean.

While we and others have previously demonstrated the effect of CLPTM1L on chemosensitivity in pancreatic cancer both in vitro and in vivo, we endeavored to investigate the hypothesis that CLPTM1L that is either excreted from tumor cells or present in shed vesicles may confer intercellular survival and chemoresistance effects in a variety of tumor types (lung, ovarian, and pancreatic). In human A549 lung tumor cells expressing cell-surface CLPTM1L (Fig. 3a), treatment with supernatants from cells that had been pre-treated with cisplatin conferred protection from cisplatin toxicity, regardless of whether the cells producing the supernatant were wild-type or CLPTM1L knockdown cells (Fig. 3b, left). However, in cells depleted of CLPTM1L by knockdown, cytoprotection from cisplatin was dependent on CLPTM1L expression in the supernatant-producing cells (Fig. 3b, right). Depletion of CLPTM1L and Akt phosphorylation at T308 and cleavage of Caspase-3 upon treatment of A549, NCI-H520, and NCI-H226 lung cancer cell lines with 102-5 anti-CLPTM1L are shown in Supplementary Fig. 5a–c. Likewise, conditioned supernatants from cells overexpressing CLPTM1L (Panc1-CLPTM1L) were protective against 100 nM gemcitabine killing compared to control supernatants with vector alone (Panc1-Vec) (Fig. 3c). The cytoprotective effect was greatly enhanced with supernatants from cells that were pre-treated with gemcitabine regardless of CLPTM1L overexpression. Depletion of CLPTM1L and Akt phosphorylation at T308 and cleavage of Caspase-3 upon treatment of Panc1, MiaPaca2, and Capan2 pancreatic cancer cell lines with 102-5 anti-CLPTM1L are shown in Supplementary Fig. 5d–f. Human ovarian tumor cells (A2780) also demonstrated resistance to carboplatin conferred by conditioned media from carboplatin-treated cells (Fig. 3d).

### Transfer of resistance by exosomal CLPTM1L

**Fig. 4:**
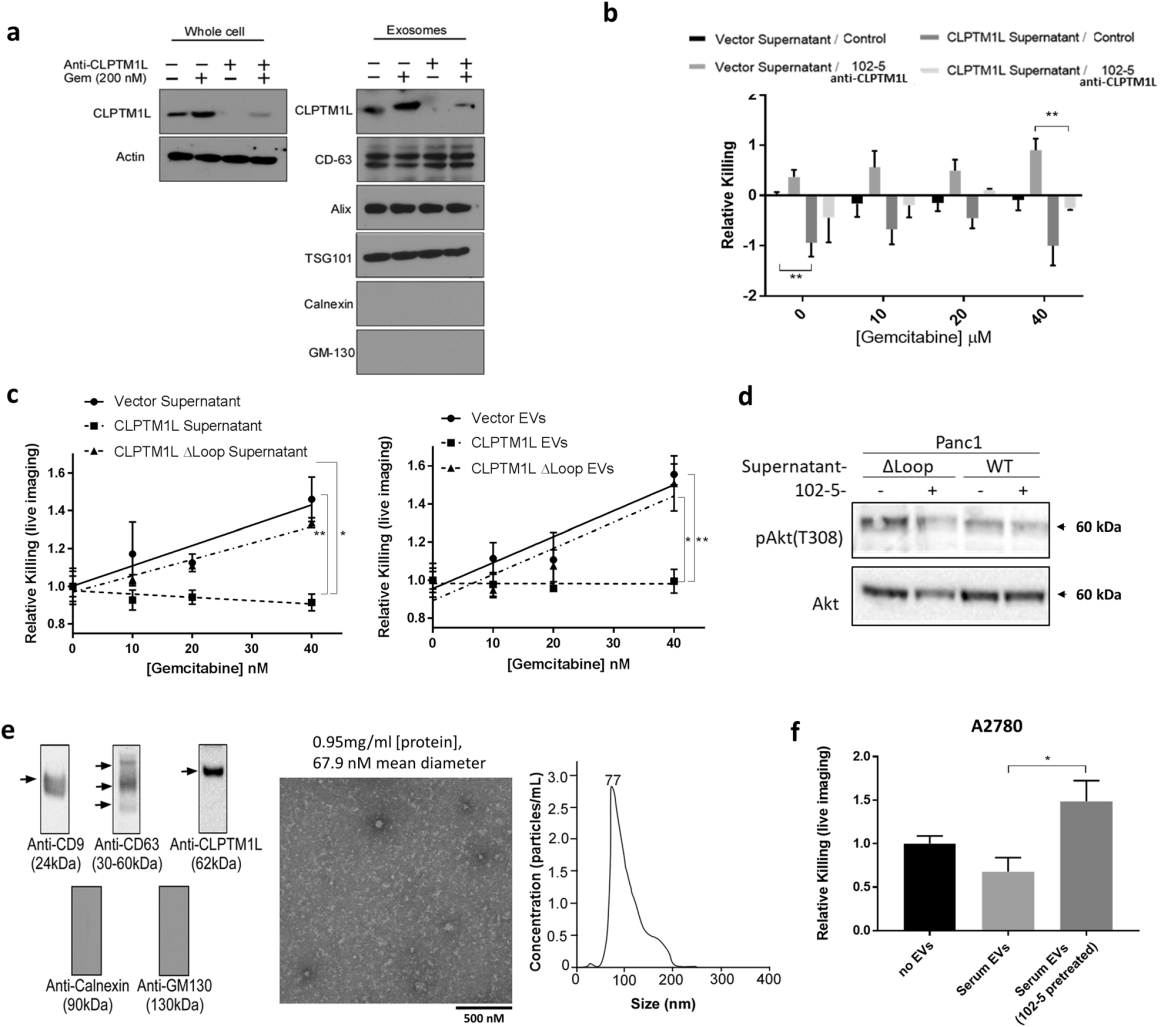
Chemo-induction and chemoprotection by extracellular vesicle- and supernatant-associated CLPTM1L ectodomain. **a** Western blotting for CLPTM1L in whole-cell lysates and exosomes isolated from Panc1 cells treated with vehicle control, anti-CLPTM1L and/or 200 nM gemcitabine. CD63, Alix and TSG101 serve as exosomal markers and Calnexin and GM-130 serve as ER and golgi markers, respectively. **b** Relative killing (live imaging cytotoxicity) of Panc1 cells treated for 48 h with 40 nM gemcitabine and culture supernatants from donor cells with vector control or overexpressing CLPTM1L. Error bars represent standard error of the mean. **c** Relative killing (live imaging cytotoxicity) of Panc1 cells treated with 0–40 nM gemcitabine and culture supernatants (1:1 mix with fresh media) (left panel) or 25 µL extracellular vesicle (EV)-containing (ExoQuick) fraction per mL of media (right panel) from Panc1 donor cells with vector control, CLPTM1L overexpression, or CLPTM1L ectodomain deletion mutant (CLPTM1L ΔLoop) overexpression. Error bars represent standard error of the mean. **d** Western blotting of Panc1 lysates treated as above with the indicated supernatants and/or 102-5 anti-CLPTM1L for phospho-Akt (T308) and total Akt. **e** Characterization of extracellular vesicles isolated from human serum by ultra-centrifugation by western blotting for exosomal markers CD9 and CD63, non exosomal markers Calnexin and GM130 and CLPTM1L using 102-5 anti-CLPTM1L antibody (left); and by transmission electron microscopy (center). Scale bar = 500 nM. Nano-sight results demonstrating size-distribution and concentration of exosomes from culture supernatants (right). **f** Relative killing of A2780 ovarian tumor cells treated with either vehicle control, human serum extracellular vesicles, or human serum extracellular vesicles pretreated for 24 h with 200 nM 102-5 Anti-CLPTM1L, and then treated with 40 µM carboplatin for 4 days. Error bars represent standard error of the mean. *p < 0.05.

Extracellular vesicle preparations containing exosomes from culture supernatants of OVCAR5-Cis^R^ contained CLPTM1L, but that from OVCAR5 cisplatin sensitive cells did not (Fig. 3e). The presence of CLPTM1L in isolates from supernatants was decreased by treatment with 102-5 anti-CLPTM1L and undetectable when the exosome production inhibitor DMA was added to cells. The disappearance of exosomes in these fractions upon DMA treatment was confirmed by the disappearance of exosomal markers CD-63, Alix, and TSG-101 (Fig. 3e). Given these findings, we sought to determine if exosomal CLPTM1L could confer cisplatin resistance to sensitive tumor cells. Treatment with OVCAR5-Cis^R^ conditioned media inhibited cisplatin killing in sensitive OVCAR5 cells (Fig. 3f). Treatment with either 102-5 anti-CLPTM1L or exosome production inhibitor DMA restored cisplatin sensitivity in cells with conditioned media. Similarly, Treatment with HeyA8-Cis^R^ conditioned media inhibited cisplatin killing in sensitive HeyA8 cells (Supplementary Fig. 6). Again, treatment with either 102-5 anti-CLPTM1L or exosome production inhibitor DMA restored cisplatin sensitivity in cells with conditioned media. Investigating multiple tumor types, we found that CLPTM1L was present in the extracellular vesicle fractions culture media from Panc1 pancreatic and cisplatin resistant variants of ovarian tumor cells (Figs. 3e and 4a and Supplementary Fig. 6). The exosomal production inhibitor DMA depleted the presence of both exosomal markers and CLPTM1L in these fractions (Supplementary Figs. 6 and 7). The abundance of CLPTM1L in the exosomal/extracellular vesicle fraction from Panc1 cells was increased upon treatment of cultures with gemcitabine (Fig. 4a). The size distribution and concentration of exosomes/extracellular vesicles isolated from OVCAR5 culture supernatant is depicted in Fig. 4e and is representative of exosome isolates. Treatment of human pancreatic tumor cells with full-length human anti-CLPTM1L 102-5 resulted in sensitization to gemcitabine killing. 102-5 also abrogated the cytoprotection conferred by supernatants, particularly those from CLPTM1L-overexpressing cells (Fig. 4b). This cytoprotection was ablated by pre-treatment of the conditioned supernatants with human anti-CLPTM1L 102-5.

Cytoprotection from gemcitabine killing by either culture supernatants (Fig. 4c, left panel) or ExoQuick preparations containing extracellular vesicles (Fig. 4c, right panel) from CLPTM1L overexpressing Panc1 cells was dependent on the ectodomain of the exogenously expressed CLPTM1L (Fig. 4c). Regardless of the presence of the ectodomain, inhibition of CLPTM1L using 102-5 anti-CLPTM1L resulted in decreased Akt phosphorylation at threonine 308 (Fig. 4d). Panc1 cells treated with supernatants from cells overexpressing wild-type CLPTM1L were protected from gemcitabine killing at concentrations up to 40 nM, while those treated with no supernatant or supernatants from vector or CLPTM1L exodomain deletion mutant (CLTPM1L ΔLoop) exhibited statistically similar dose-dependent gemcitabine killing. Results demonstrating the effect of extracellular CLPTM1L on chemosensitivity are summarized in Table 1. Extracellular vesicles isolated from human serum by ultracentrifugation and characterized by CD9 and CD63 expression as exosomal markers and Calnexin and GM130 as a non-exosomal markers and transmission electron microscopy contained CLPTM1L as detected by western blot (Fig. 4e). The size distribution and concentration of extracellular vesicles isolated from OVCAR5 culture media is also demonstrated by the representative results using Nano-sight methodology in Fig. 4e (right). Carboplatin killing of A2780 ovarian tumor cells treated with serum extracellular vesicles trended lower compared to those not treated with extracellular vesicles (Fig. 4f). Pre-treatment of serum extracellular vesicles with 102-5 anti-CLPTM1L abrogated resistance to carboplatin with significantly increased killing compared to vesicles that were not pre-treated (Fig. 4f). Extracellular vesicles were isolated using two different methods for these experiments, increasing confidence that the observed effects were not due to non-vesicle contaminants.

**Table 1:** Summary of chemosensitivity data from Figs. 3 and 4.

	Figure panel-	3b left				3b right				3c		3d			4b				4c		
Target Cells	Vector/wild-type	X	X	X	X					X	X	X	X	X	X	X	X	X	X	X	X
	CLPTM1L knockdown					X	X	X	X												
Donor Cells	Vector	X	X			X	X			X					X	X			X		
	CLPTM1L knockdown			X	X			X	X												
	CLPTM1L overexp.										X						X	X		X	
	CLPTM1L ∆loop overexp.																				X
Supernatant Pre-treatment	Control	X		X		X		X				X									
	Chemo (Gem or Carbo)		X		X		X		X				X	X							
	anti-CLPTM1L													X							
Target Cell Treatment	Control														X		X				
	anti-CLPTM1L															X		X			
Chemoresistance			+**+**	−	+		+/−	−	−	−	+	−	+	−	+	−	++	+	−	+	−

Panel 3b data are expressed as relative to shaded control columns. Chemoresistance = statistically significant decrease in chemotherapeutic killing. (X)s indicates the conditions or phenotypes for each column as defined by labels in the left two columns.

### Anti-CLPTM1L inhibition of disseminated ovarian tumorigenesis in a syngeneic-orthotopic model and xenograft models

**Fig. 5:**
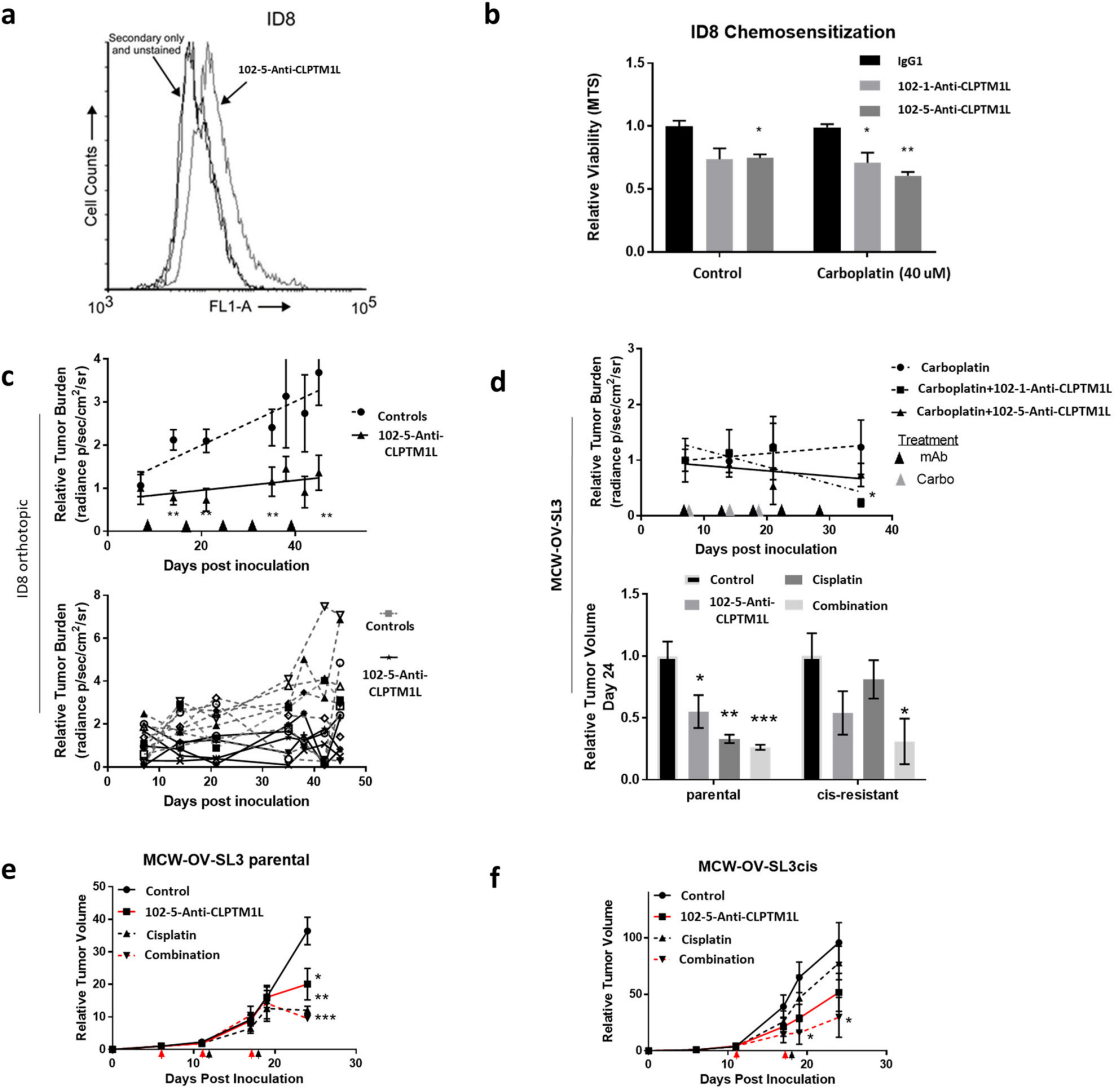
Orthotopic syngeneic isograft and human xenograft ovarian cancer models. **a** Flow cytometry histograms of fluorescent labeling of live ID8-luc cells with 102-5 anti-CLPTM1L primary and Alexafluor-488-conjugated secondary antibodies. **b** Viability of ID8-luc cells treated with 100 nM 102-5 anti-CLPTM1L and/or 40 µM carboplatin for 5 days. Error bars represent standard error of the mean. **c** Relative tumor burden as measured by luciferin luminescence live animal imaging of control vs. 10 mg/kg 102-5 anti-CLPTM1L treated ID8-luc isograft groups (top panel) and individual animals (bottom panel). Error bars represent standard error of the mean. **d** Relative tumor burden as measured by luciferin luminescence live animal imaging of mice treated with 10 mg/kg carboplatin plus either control, 102-1 or 102-5 anti-CLPTM1L (10 mg/kg). Error bars represent standard error of the mean. **e** Final relative mean volumes of MCW-OV-SL3 and MCW-OV-SL3-Cis^R^ xenograft tumors 24 days post-inoculation. Error bars represent standard error of the mean. **f** Mean volumes of MCW-OV-SL3 and MCW-OV-SL3-Cis^R^ xenograft tumors relative to group mean volume after sorting from days 0 to 24 post-inoculation. *p < 0.05, **p < 0.005, ***p < 0.0005. Error bars represent standard error of the mean.

Having demonstrated sensitization of tumor cells to genotoxic chemotherapeutic agents and tumoricidal activity by CLPTM1L inhibition, and with a lack of in vivo evidence in ovarian cancer models, we sought to determine if anti-CLPTM1L mAb treatment could inhibit ovarian carcinoma in relevant murine models, including a cisplatin resistant tumor model. With disseminated peritoneal disease being the most common presentation upon recurrence of ovarian carcinoma, we utilized an orthotopic isograft model of disseminated peritoneal ovarian cancer. Human anti-CLPTM1L binding to luciferase-expressing ID8 ovarian tumor cells (ID8-luc) was detected by flow cytometry (Fig. 5a). Treatment of ID8-luc cells in vitro resulted in significantly decreased viability (ANOVA *p* < 0.0001) and increased carboplatin killing (ANOVA *p* < 0.05) over 5 days in culture (Fig. 5b). ID8-luc cells were injected intraperitoneally into syngeneic C57bl/6 mice. Tumor load was monitored by luciferin luminescence using whole body imaging. Treatment of mice (weekly, I.P.) with 10 mg/kg 102-5 anti-CLPTM1L resulted in inhibition of tumor growth over 8 weeks (Fig. 5c). Control mice had an average increase in tumor load of 2.45-fold at day 42, while 102-5 anti-CLPTM1L-treated mice had an average of 0.90-fold relative tumor load at day 42 (*T*-test *p* < 0.005) (Paired *t*-test across time points, *p* = 0.001). This anti-tumorigenic activity was similar to that of 10 mg/kg carboplatin administered weekly for three consecutive weeks (Fig. 5d). With combination treatments of carboplatin following anti-CLPTM1L treatment, tumor load was decreased (0.23-fold) over 35 days compared to mice treated with carboplatin alone, in which tumor load increased slightly by an average of 1.23-fold (*T*-test *p* < 0.05) (Fig. 5d).

In a complementary model of platinum resistance, J/NU mice xenografted with MCW-OV-SL3 parental or MCW-OV-SL3-Cis^R^ platinum resistant cells were treated weekly with control vehicles, 2.5 mg/kg cisplatin, and/or 5 mg/kg 102-5 anti-CLPTM1L. The effects of cisplatin and those of 102-5 anti-CLPTM1L on cisplatin treatment were investigated in this model rather than those of carboplatin since the MCW-OV-SL3-Cis^R^ line was developed specifically as a cisplatin resistant line. Tumor growth and volumes, monitored over 24 days post-inoculation, were significantly inhibited by both cisplatin and 102-5 anti-CLPTM1L either as individual monotherapies or as a combination (Fig. 5e, and f). In MCW-OV-SL3-Cis^R^ platinum resistant xenografts, cisplatin significantly inhibited tumor growth only in combination therapy with 102-5 anti-CLPTM1L.

## Discussion

The identity of CLPTM1L as a true oncofetal protein is gaining credence with the accumulation of knowledge regarding the pathologic function of the protein and recent evidence of fetal development function in genetically engineered CLPTM1L knockout mice^13^. CLPTM1L anti-apoptotic properties may be critical during fetal development as both CLPTM1L and family member CLPTM1 have been implicated in cleft lip and palate syndrome^19,20^. In adult animals, cell-surface expression of CLPTM1L is restricted to iPS, ePS, and malignant cell populations^14,21^. Numerous consortia have demonstrated common overexpression of CLPTM1L in a variety of tumor types^2,10–13,15,16,22^. Data collected and annotated by the Human Protein Atlas also demonstrates high CLPTM1L expression in a variety of cancers including ovarian serous adenocarcinoma with no expression in normal ovary tissue or normal fallopian epithelium (available from http://www.proteinatlas.org). High expression of CLPTM1L and CLPTM1L expression-controlling polymorphisms^23^ in tumors are associated with poor prognosis as described here and previously in^24^. The function of CLPTM1L is best understood in the context of anti-apoptotic activity^2,10–12,16,17,25^.

Restriction of CLPTM1L surface expression to stem cell populations^14,21^ and tumor cells presents the potential to target quiescent residual tumor cells and to eliminate inherent or acquired resistance to multiple chemotherapeutic drugs. Notably, treating platinum resistant ovarian tumor cell lines with 102-5 anti-CLPTM1L resulted in depletion of cancer stem cell markers and a corresponding sensitization to cisplatin. Depletion of these markers of stemness and inhibition of tumor spheroid growth suggest that 102-5 anti-CLPTM1L may prevent survival of cancer stem cells or stemness-related programming. Therefore, CLPTM1L inhibition may be an attractive approach to adjunctive and/or maintenance therapy in ovarian and other cancers. Here we have demonstrated robust inhibition of tumorigenesis and sensitization of tumors to chemotherapeutic killing using anti-CLPTM1L monoclonal antibodies, which can also be achieved using shRNA depletion^10,11^. While tumor burden was nearly undetectable at endpoint in ID8 syngeneic isograft models with combination treatment, this does not preclude the possibility of tumor regrowth. Long-term, high-dose curative therapy models are an important subject of future investigation. The restoration of platinum sensitivity in patient-derived cisplatin-resistant xenografts as demonstrated in this study is notable. In the present study, we demonstrate that CLPTM1L accumulation is upregulated in platinum resistant derivatives of human ovarian tumor cells. This was concurrent with higher Akt phosphorylation, and 102-5 anti-CLPTM1L significantly decreased Akt phosphorylation. ShRNA depletion of CLPTM1L also decreases Akt phosphorylation, which is mechanistically linked to oncogenic transformation^10^, indicating that decreased Akt phosphorylation in 102-5 anti-CLPTM1L-treated cells and tumors is not due to off-target effects. Targeted therapies such as PARP and VEGF inhibitors are currently being used in ovarian cancer in the adjuvant setting and have been recently investigated in a maintenance therapy setting, although PARP inhibition may only be effective in a small subset of patients, for example, those with homologous DNA damage repair defects (BRCA). VEGF inhibition has exhibited a modest effect on overall survival. We and others have published an overabundance of data in pancreatic and lung cancer cells on the effects of CLPTM1L inhibition in in vitro and in vivo assays for chemosensitization and the mitigation of drug resistance^2,10–12,16,17^, as we now describe herein for ovarian cancer. Therefore, we have not included those cancer types in those aspects of the study. However, the extracellular effects demonstrated in ovarian cancer have not been previously seen in other cancers. We have therefore included pancreatic and lung cancer cells as part of a larger study on intercellular transfer of chemoresistance through CLPTM1L. Potentiation of genotoxic killing of tumors using chemotherapy drugs such as carboplatin using CLPTM1L inhibitors may be practical approach to mitigating therapy resistance in ovarian cancer. Equal protection from genotoxic killing by CLPTM1L supernatants and extracellular vesicles fractions in a CLPTM1L ectodomain-dependent manner is strong evidence that CLPTM1L in extracellular vesicles is indeed responsible for an observed transfer of cytoprotection by supernatants from CLPTM1L-expressing cultures. Treatment of tumor cells with supernatant from wild-type CLPTM1L overexpressing cells did not cause a detectable increase in phosphorylation of Akt compared to treatment with supernatants from ΔLoop CLPTM1L overexpressing cells. However, inhibition of CLPTM1L decreased Akt phosphorylation in cells treated with both wild-type CLPTM1L and ΔLoop CLPTM1L. This suggests that while endogenous CLPTM1L may mediate chemoresistance through Akt phosphorylation in ovarian tumor cells including A2780, HeyA8, OVCAR5, PeO4, Akt phosphorylation may be a less prominent CLPTM1L-mediated chemoresistance mechanism when it is delivered from extracellular sources.

Tumor-derived extracellular vesicles have been previously shown to mediate resistance to chemotherapy in bystander cells, including in ovarian cancer^26–28^. Exosomes were found to promote an EMT phenotype, which may contribute to resistance^26^. Interestingly, CLPTM1L interacting protein and ER-stress survival effector GRP78 can promote EMT in cancer^29^. The role of plasma membrane or extracellular CLPTM1L in EMT in cancer is a provocative subject of further research. While increased CLPTM1L in the extracellular vesicles fraction of treated patients may be the result of generally increased vesicle shedding by tumor cells, we have demonstrated the concept that extracellular CLPTM1L can transfer chemoresistance. There are clearly secreted factors other than CLPTM1L that are induced by chemotherapeutic insult, as demonstrated by cytoprotection of CLPTM1L expressing cells by both vector control and CLPTM1L shRNA supernatants in (Fig. 3b). We have shown that in tumor cells depleted of CLPTM1L, intercellular CLPTM1L from supernatants becomes important for cytoprotection against chemotherapeutic toxicity. The absence of an effect of anti-CLPTM1L on CLPTM1L abundance in extracellular vesicles may be due to internalization and incorporation of CLPTM1L into endosomes upon mAb treatment.

## Methods

### Cell culture and reagents

OVCAR4, HeyA8, PeO1, PeO4 Panc1, MiaPaCa2, CaPan2, A549, H226, and H520 human tumor cells were obtained from ATCC. A2780 parental and cisplatin resistant A2780cis cells were obtained from Millipore Sigma (St. Louis, MO). OVCAR5 cell line was purchased from NCI-DCTD repository and authenticated within 6 months of experiments. Fallopian tube epithelial cells FTE187 and FTE188 were received from Jinsong Liu at MD Anderson Cancer Center, Houston, TX, USA. Immortalized human pancreatic stellate (HPSC)—a cancer-associated fibroblasts cell line was used as defined previously^30^. All cell lines were cultured in recommended medium under standard conditions and were examined for mycoplasma contamination by mycoplasma PCR detection kit (Applied Biological Materials Inc., Richmond, Canada) as describe previously^31,32^. ID8 cells expressing luciferase were obtained from the Katherine Roby Laboratory at the University of Kansas and were authenticated within 6 months of experiments (RRID: CVCL_IU15). Gemcitabine, carboplatin and DMA were purchased from Sigma-Aldrich (St. Louis, MO). Polyclonal anti-CLPTM1L (HPA014791, Sigma Aldrich, St. Louis, MO) or monoclonal anti-CLPTM1L antagonist^12^ was used in anti-CLPTM1L inhibition studies. Human ovarian epithelial carcinoma tissue obtained as per MCW-IRB-Human Research guidelines and verified as endometrioid subtype of ovarian cancer was used to develop MCW-OV-SL-3 cell line, which was cultured in DMEM 10%fetal bovine serum, 50 U/mL of penicillin/streptomycin, 2 mmol/L of L-glutamine, 1 mmol/L of sodium pyruvate and were grown at 37 °C in an atmosphere of 5% carbon dioxide. These cells were validated by STR sequencing (IDEXX BioAnalytics, using CellCheck 16 Plus profile) and found to be unique and distinct from any known cell line. Cisplatin resistant lines (OVCAR5-CisR, HeyA8-CisR, and MCW-OV-SL3-CisR) were established by exposure to increasing concentrations of cisplatin over a period of 12 months. For CLPTM1L heterozygous knockout as in supplementary data, Guide RNAs were selected for CRISPR KO of hCLPTM1L. HEK293T cells were co-transfected with linear DNA containing a fluorescent marker and a puromycin selection cassette and a pCas-Guide vector containing Cas9 and sgRNA comprising either scrambled sequence or human CRR9 specific shRNA. Transfected HEK293T cells were determined to be GFP positive by flow cytometry and were puromycin resistant after selection on puromycin 10 µg/mL for 10 days and maintenance on puromycin 1 µg/mL. Possible off target mutation was analyzed using CHOPCHOP. Guide RNAs had no unintended targets with ≤2 mismatches and minimum efficiencies of 58%.

Antibody diluent as described by Abcam was used as a vehicle control for polyclonal antibody treatment where indicated to account for any effect of diluent constituents. Normal mouse IgG (Thermo Fisher, 02-7102) was used as a non-specific antibody control for monoclonal antibody treatment where indicated. Rabbit α-HA (Santa Cruz Biotechnology, Santa Cruz, CA. Y-11) was used as a non-specific antibody control for polyclonal antibody treatment where indicated. Mouse α-HA (Cell Signaling, Boston, MA, 6E2) was used as a non-specific antibody control for experiments with purified monoclonal antibodies, and mouse α-human Von Willebrand Factor (hVWF) ascites was used for experiments with monoclonal ascites. Monoclonal antibody design, production, and evaluation were done in collaboration with Essential Biotechnology, LLC. (Big Bend, WI). Mouse hybridoma work was contracted to Biomatik Corporation, Cambridge, Ontario, Canada. Expression constructs for FLAG-tagged wild-type and ΔLoop CLPTM1L were generated and transfected as described in ref. ^15^. Human monoclonal antibody inhibitors of CLPTM1L were derived from panning results of a naïve human phage display library (Neoclone, Madison WI) against CLPTM1L peptide antigen representing an ectodomain epitope as defined in^12^. Resulting flag tagged scFv clones used in this study are 102-1b, 102-4a, and 102-5a. Heavy and light chain variable regions from clones 1b and 5a were cloned into expression vectors in a full-length human IgG1 framework and expressed transiently in Chinese hamster ovary cells 102-1 and 102-5 (Catalent, Madison WI). 102-5 was selected as a lead based on affinity and on-target potency in chemosensitization and anchorage independent growth inhibition.

### Immunohistochemistry

A full FDA panel of normal human tissues on tissue microarray (TMA) (H1.0 × 144) was obtained from Proteogenex (Inglewood, CA). A panel of tumor tissue sections from 24 ovarian cancer (high-grade serous adenocarcinoma) patients was obtained from the MCW Tissue Bank. Human patient-derived xenograft tissue slides (FFPE) were obtained from Jackson Labs. IHC staining for CLPTM1L (Human: Polyclonal anti-CLPTM1L (HPA014791, Sigma Aldrich, St. Louis, MO, Mouse: Novus anti-CLPTM1L, NBP1-62477) was performed on the Dako Autostainer Plus using the Dako EnVision™ FLEX High pH Detection Kit (K8010) (Dako, Carpinteria, CA). Immunohistochemical staining performed as described earlier^33,34^. Briefly, slides were deparaffinized to Xylene. Antigen Retrieval was performed on Dako PT Link water bath. The antigen retrieval was done at 97 °C for 20 min. The slides were cooled until they reached 65 °C. All slides for all antibodies were placed in Tris/EDTA pH 9 (Dako TRS High pH). Slides were washed in Dako wash buffer for 5 min. Slides were subjected to a peroxidase Block for 5 min. Slides were rinsed twice with wash buffer. Slides were incubated with primary antibody CLPTM1L (rabbit polyclonal, Sigma Aldrich cat# HPA014791, lot A57952) diluted to 1:400 for 30 min. Slides were rinsed with wash buffer. Slides were incubated with secondary antibody for 20 min and rinsed twice with wash buffer. Slides were incubated with DAB substrate for 10 min and rinsed with wash buffer. Slides were stained with hematoxylin for 7 min and rinsed with DI water. Slides were dehydrated and mounted for viewing. Omission of the primary antibody served as negative control. Upon pathologic review, staining intensity in each tumor tissue was scored in triplicate and averaged. Tumors were scored as negative—0, weak—1, intermediate— 2, or strong—3. Independent scores were averaged. A staining index was calculated by multiplication of staining score and percentage of tumor cells with that score.

### Western blotting

Protein expression was accessed by western blotting as discussed earlier^35^. Briefly cells were lysed with 100 μL of 1× NP40 lysis buffer containing proteinase inhibitors, sheared 10 times with a 28-gauge needle, spun at 16,000 × *g* for 30 min, normalized by protein concentration as determined by the Bradford method, and the supernatant boiled for 5 min. Twenty microliters of normalized lysate were resolved by SDS-PAGE on 10% acrylamide gels at 150 volts for 1 h, transferred by iBlot dry transfer (Life Technologies), blocked for 2 h with TBST 10% milk, incubated with primary antibodies at 1:1000 in TBST 5% milk at 4 C overnight with rocking, washed 3 × 5 min with TBST, incubated with secondary anti-rabbit-HRP (Santa Cruz Biotechnology) or goat anti-human-HRP (Invitrogen MH1715) at 1:10,000 in TBST 5% milk for 2 h with rocking, washed 5 × 10 min with TBST, and signal analyzed using a Biorad Chemidoc XRS. The following antibodies were used: rabbit anti-CLPTM1L (HPA014791, Sigma-Aldrich, St. Louis, MO) (human) or (NBP1-62477, Novus Biologicals, Littleton, CO) (Mouse), anti-Actin (Santa Cruz Biotechnology, 58673), anti-AKT (9272), anti-pAKT (Thr308) (9275), all at a dilution of 1:1000. Quantitation of Western blot analyses of three independent cultures was done using Image J software. All blots derive from the same experiment and were processed in parallel.

### Live cell imaging

Cells were plated on 24-well tissue culture plates at equal density (~80) confluence and allowed to attach overnight before treatment as indicated in at least triplicate. Culture media contained yoyo-1 fluorescent DNA intercalating dye (ThermoFisher) at a 1:10,000 dilution. Confluence and fluorescent dead cell counts per well were monitored at 3 h intervals using an Incucyte FLR live cell imager and software (Essen Biosciences, Ann Arbor, MI). Student’s T-test determined significance of differences between groups, and ANOVA determined significance of interaction between treatments.

### Cytotoxicity assays

Cells were plated at equal density on 24-well tissue culture plates and treated as indicated. Cells were treated with monoclonal anti-CLPTM1L for 24 h followed by 72 h of treatment with gemcitabine at the indicated concentrations. Dead cells were fluorescently stained with Yoyo-1 (Life Technologies) and enumerated on an Incucyte FLR live cell imager (Essen Bioscience). Total cell numbers were then enumerated on the imager by staining with Vybrant Dye Cycle Green (Life Technologies) or by permeabilization to Yoyo-1 with 0.01% Triton X-100. A killing index was calculated for each well by dividing the number of dead cells by the number of total cells. The killing indices of triplicate groups were averaged.

### MTT cell viability assays

To measure cell viability, the MTT assays were performed as described earlier^36^. Briefly, cisplatin resistant cells and their parental cells were plated at a density of 1 × 10^4^ cells in 96 well plates after treatment with cisplatin at varying concentrations with and without 102-5 anti-CLPTL1L antibody. 3-(4,5-dimethylthia- zol-2-yl)-2,5-diphenyltetrazolium bromide (MTT, Sigma Aldrich, MO, USA) reagent was added and incubated at 37 °C for 4 h. This was followed by dissolving formazan crystals with acidic isopropanol. Absorbance was measured in microplate reader (Tecan, Mannedorf, Switzerland) at 560 nm. IC50 was calculated using Graph Pad Prism software.

### Treatment of cells with conditioned media

Fresh media was placed on 10 cm tissue culture dishes of sub-confluent tumor cells (donor cells), which were then treated as indicated. carboplatin-conditioned supernatants were collected after 48 h of treatment and gemcitabine pre-treated-cell supernatants were collected after 72 h of treatment. Media was changed and then collected after 48 h of additional incubation, spun at 400 × *g* for 5 min to pellet cells, and mixed with fresh media at a 1:1 ratio. Anti-CLPTM1L pre-treatment of conditioned supernatants was performed at 4 °C overnight. The supernatant/media mixture was pretreated with antibody as indicated and/or added to cells in multi-well plates. Cells were treated with chemotherapeutic agents (Gemcitabine or Carboplatin, Sigma Aldrich) as indicated, 2.5–4 h after treatment with supernatants. Live cell imaging as described above was used to longitudinally monitor cytotoxicity after treatment. Endpoint data was taken from 12 to 60 h post treatment as indicated.

### Isolation of extracellular vesicles from serum and culture media

Isolation by Ultracentrifugation Protocol: De-identified serum was obtained from the MCW Tissue Bank and Surgical Biorepository from PDAC and serous ovarian adenocarcinoma patients. The de-identified samples were determined not to fall under human subjects research by the MCW Institutional Review Board. One milliliter of serum was centrifuged at 2000 × *g*, 4 °C, 15 min on a tabletop centrifuge to remove debris. Supernatant was centrifuged at 10,000 × *g*, 4 °C, 30 min on a tabletop centrifuge to remove microvesicles. Supernatant was ultracentrifuged with a SW-55Ti rotor; 140,000 × *g* (34,000 rpm); 4 °C; overnight (18 h) with a 25% sucrose cushion. The pellet was washed 3 times with PBS and ultra-centrifuged at 140,000 × *g*, 4 °C, 30 min and resuspended in 100 µL PBS, stored at −20 °C. Vesicle size was measured using AMT Image Capture Engine Software Version 602.571. Protein concentration of samples was measured on a Qubit (Life Technologies). Twenty microliters were added to 10 mL of culture media for treatment of cells.

Cell culture supernatants (~10 mL) were collected from donor cells, centrifuged at 300 × *g* for 10 min at 4 °C to eliminate cells and cellular debris. Supernatants were then centrifuged at 2000 × *g* for 20 min at 4 °C. One mL of Exoquick TC (System Bioscience, Palo Alto, CA) was added to the remaining supernatant and incubated overnight at 4 °C. The mixture was centrifuged at 1500 × *g* for 30 min at 4 °C. The pellet containing extracellular vesicles was suspended in 50–500 µL of RIPA with Halt protease cocktail and stored at −20 °C. Where indicated, sub-confluent cells were treated with 200 nM 102-5 anti-CLPTM1L for 24 h before adding 200 nM gemcitabine and incubating for an additional 48 h. Extracellular vesicles were then isolated from culture supernatants and whole-cell lysates were obtained. The size and concentration of exosomes purified from cell culture supernatants or patient’s plasma were determined using NanoSight N 3000 (Malvern Instruments). Exosome-depleted FBS (Gibco, Thermo Fisher Scientific Inc., MA, USA) was used for all exosome-related experiments.

### Flow cytometry

Flow cytometry was conducted as described previously^37,38^. Tumor cells were harvested by trypsinization and suspended in cold PBS, 10% FBS, .05% NaN_3_. 100 µL of cell suspension was added to 12 × 75 mm polystyrene tubes. 10 µg/mL of primary antibody diluted in PBS 3% BSA was added and tubes were incubated at 4 °C in the dark for 45 min. After washing 3 times in cold PBS, cells were resuspended in rabbit anti-Human Fc (H&L)-Alexafluor 488 (Abcam) in PBS 3% BSA and incubated at 4 °C in the dark for 30 min. After washing 3 times in cold PBS, cells were suspended in 500 µL cold PBS, 3% BSA, 0.05% NaN_3_ and analyzed on an Accuri C6 flow cytometer. Cells were gated for live cells based on scatter and fluorescence was plotted on a histogram using Flowing Software 2 (Turku Bioimaging).

### Phage display screening, codon optimization, and expression of scFv, scFv-Fc, and fully human anti-CLPTM1L

Phage display library screening against CLPTM1L peptide antigen was conducted by Neoclone, LLC, Madison WI. All positive binders in selection and enrichment that were also negative to appropriate counter-screens were analyzed by sequence analysis for identification of unique clones. Individual unique clones were tested for specificity by phage ELISA against the target antigen and carried forward if confirmed. Codon optimization and expression as scFv-Fc fusions by transient transfection of cloned vectors into 293T cells was conducted by Lytic Solutions, LLC in Madison WI. Cloning and expression of full-length human IgG1 in Chinese hamster ovary cells was conducted by Catalent Pharma Solutions in Madison WI.

### Ovarian tumor models

#### ID8-luc model

Five-week-old C57bl/6 mice (Jackson labs strain 664) were acclimated for one week and injected intraperitoneally with 5 × 10^5^ luciferase expressing ID8 syngeneic ovarian tumor cells suspended in 200 µL of PBS under Medical College of Wisconsin animal protocol AUA6339. One week after injection, antibodies and/or chemotherapeutic agents were administered intraperitoneally at the indicated doses. Chemotherapeutic doses or vehicle were administered one day before control or therapeutic antibody doses. Imaging and analysis of luciferin radiance was done up to twice weekly on an IVIS Spectrum-CT Imaging System equipped with thermoelectrically-cooled CCD camera. Mice were monitored twice weekly for weight and signs of morbidity. Mice were euthanized after 45 days or upon signs of morbidity. Animal studies were conducted using the standards for humane treatment of animals as described in the U.S. Public Health Service Policy on Humane Care and Use of Laboratory Animals.

### MCW-OV-SL3 xenograft model

Four-week-old female NU/J mice were purchased from Jackson Laboratories and housed in pathogen free conditions. All in vivo experiments and protocols were reviewed and approved by MCW IACUC committee. To establish the tumors, MCW-OV-SL3 or cisplatin-resistant version of cells (0.5 × 10^6^ cells/mouse) were injected into the flank region subcutaneously. After tumors had grown to an average of 100 mm^3^, mice were separated randomly into groups of 5 mice. Mice were treated weekly with intraperitoneal doses of PBS control, cisplatin 2.5 mg/kg, and/or Antibody 102-5 clone 5 mg/kg in 200 µL of PBS at room temperature beginning on day 6 post-inoculation. Cisplatin treatments were administered 24 h following antibody treatment. Tumor volume was measured every 3 days to weekly.

Animal health was monitored daily and animal weights measured at least weekly. All procedures were reviewed and approved my MCW IACUC committee #2 under AUA6339.

### Spheroid growth and viability assays

For spheroid formation, cancer cells (1000/well) were plated in 6 well chambered glass slides (LabTech) in DMEM containing 10% FBS, 1% penicillin/streptomycin, 5 ng/mL EGF, and 2% matrigel growth factor reduced (GFR) basement membrane matrix as previously described^39^. Fresh medium was supplemented every 2 days. The spheroids were counted and photographed at day 14. Spheroids were monitored every two days and images were taken on the 14th day with ×100 magnification using Nikon Eclipse TS2 Microscope. Spheroid viability was analyzed utilizing Celltiter-Glo 3D cell viability assay (Promega) according to the manufacturer’s instructions. Combination indices were calculated by $CI = \frac{{(C)1}}{{(C_X)1}} + \frac{{(C)2}}{{(C_X)2}}$ where (*C*)1 and (*C*)2 are the doses of drugs 1 and 2 with effect *x* when used together and (*CX*)1 and (*CX*)2 are the doses of drugs 1 and 2 that accomplish effect *x* when used alone.

### Plasmid construction

Residues 43–278 of the CLPTM1L ectodomain was cloned into a previously described pET28a-Smt3 expression vector^40^. The final, purified CLPTM1L ectodomain protein possessed an N-terminus starting at residue 43 of the ectodomain. The CLPTM1L ectodomain DNA sequence was confirmed by DNA sequencing.

### Protein expression and purification

The Smt3-CLPTM1L-ecotodomain expression vector was transformed into *E. coli* strain BL21 (DE3) and cells were grown at 37 °C in Luria-Bertani medium. Protein expression was induced by the addition of isopropyl-ß-D-thiogalactopyranoside when the culture reached an OD_600_ of 0.7. After incubation at 37 °C for 5 h, cells were pelleted at 5000 × *g* and stored at −80 °C. Cell pellets were resuspended in a buffer containing 50 mM sodium phosphate (pH 8.0), 300 mM sodium chloride and 10 mM imidazole at a 10 mL per liter of cell paste. Resuspended cells were lysed by three passages through a French pressure cell. Inclusion bodies containing the Smt3-CLPTM1L-ecotodomain were collected by centrifugation at 18,000 × *g* and the supernatant discarded. The inclusion body pellet was dissolved in a buffer AD (6 M guanidinium chloride, 50 mM sodium phosphate (pH 8.0), 300 mM sodium chloride and 10 mM imidazole) and the resulting solution was clarified by centrifugation at 18,000 × *g* for 30 min. The solubilized CLPTM1L-ecotodomain was applied to 8 mL Ni-IDA column (Takara Bio, Mountain View, CA) equilibrated in buffer AD at a flow rate of 4 mL/minute. After loading, the column was washed with 5 column volumes of buffer AD and the retained CLPTM1L-ecotodomain eluted using 5 column volumes of buffer BD (6 M guanidinium chloride, 50 mM sodium phosphate (pH 8.0), 300 mM sodium chloride and 500 mM imidazole). The CLPTM1L-ecotodomain was refolded by drop-wise dilution into 50 mM sodium phosphate (pH 7.6), 150 mM sodium chloride. The solution was concentrated by ultrafiltration (MWCO 10,000 kDa) and the fusion tag cleaved by incubation with Ulp1 protease at room temperature overnight. The Smt3-tag was separated from the CLPTM1L-ecotodomain using a Ni-IDA column under non-denaturing conditions in a buffer containing 50 mM sodium phosphate (pH 8.0), 300 mM sodium chloride and 10 mM imidazole. The Ni-IDA column retained the Smt3-tag and the CLPTM1L-ecotodomain was present in the column flow through. The CLPTM1L-ecotodomain containing flow through was dialyzed into a buffer containing 50 mM sodium phosphate (pH 7.6) and 150 mM sodium chloride and stored at −20 °C. Identity of the purified CLPTM1L-ecotodomain was verified by linear trap quadrupole mass spectrometry (LTQ-MS).

### On-cell western assay

102-5 anti-CLPTM1L was labeled with IR-800 CW NHS ester (Licor, Lincoln, NE) according to the manufacturer’s instructions. Panc-1 cells grown to confluence on 48-well tissue culture plates (TPP, Midwest Scientific, St. Louis, MO) with 0–500 nM anti-CLPTM1L 102-5-IR-800 in culture media with or without excess 10-2 ascites or excess purified CLPTM1L ectodomain added prior to serial dilution for a total volume of 500 µL and incubated for 1 h at 37 °C. Plates were washed 3 times with 500 µL PBS and scanned for fluorescence on an iBright FL1000 imager (Thermo Fisher).

### ELISA

The assay was performed as described earlier with some modifications^41^. Briefly Nunc Maxisorp flat-bottom plates (Thermo Fisher) were coated with a 0–200 µg/mL dilution series of CLPTM1L ectodomain in PBS pH 7.4 and incubated at 4 °C overnight. Plates were washed 3 times with TBS with 0.1% Tween 20 (TBST). A 0–500 nM dilution series of human IgG1 isotype control or 102-5 anti-CLPTM1L in TBST with 2% milk was added to the plates and incubated at room temperature for 1.5 h. Plates were washed 3 times with TBST. Two-hundred µL of secondary got anti-human IgG-Alexafluor 488 conjugate (Thermo Fisher, Catalog # A-11013) was diluted in 10 mL TBST with 2% milk and 200 µL added to each well. Plates were incubated at room temperature for 1.5 h with rocking, washed 3 times with TBST, and read on a plate reader with 490 nM and 525 nM excitation and emission wavelengths, respectively.

### Statistical analysis

All in vitro experiments were repeated at least thrice, and the results are reported as the mean ± SE. Statistical analyses were carried out using one-sided Student’s *t* test or ANOVA as described. Statistical tests are specified in figures and table legends, with significance established at *p* < 0.05. All tests were performed using Graph Pad Prism 8 (GraphPad, La Jolla, CA, USA).

### Reporting summary

Further information on research design is available in the Nature Research Reporting Summary linked to this article.

## Supplementary information

Supplementary figures and legends

reporting summary

## Data Availability

The data generated and analyzed during this study are described in the following data record: 10.6084/m9.figshare.13379873^42^. The majority of data are openly available as part of this metadata record, and are listed in the description of the metadata record in line with the figures that they underlie in the manuscript. The following data files, listed in line with the figures in the manuscript they underlie in square brackets, are not openly available due to intellectual property protection. These files may be available upon request to the corresponding author: [Supplementary Fig. 1] Supplementary Fig.: 1g.pzf, 1h.pzf, 1i.pzf; [Supplementary Fig. 4] Supplementary Fig.: 4a.pzf, 4b.pzf, 4c.pzf, 4d.pzf.
