# Supplementary material for: Targeted biologic inhibition of both tumor cell-intrinsic and intercellular CLPTM1L/CRR9-mediated chemotherapeutic drug resistance
Source: NPJ Precis Oncol. 2021 Mar 2;5:16. doi: 10.1038/s41698-021-00152-9 (PMC7925570; doi:10.1038/s41698-021-00152-9)

Supplementary

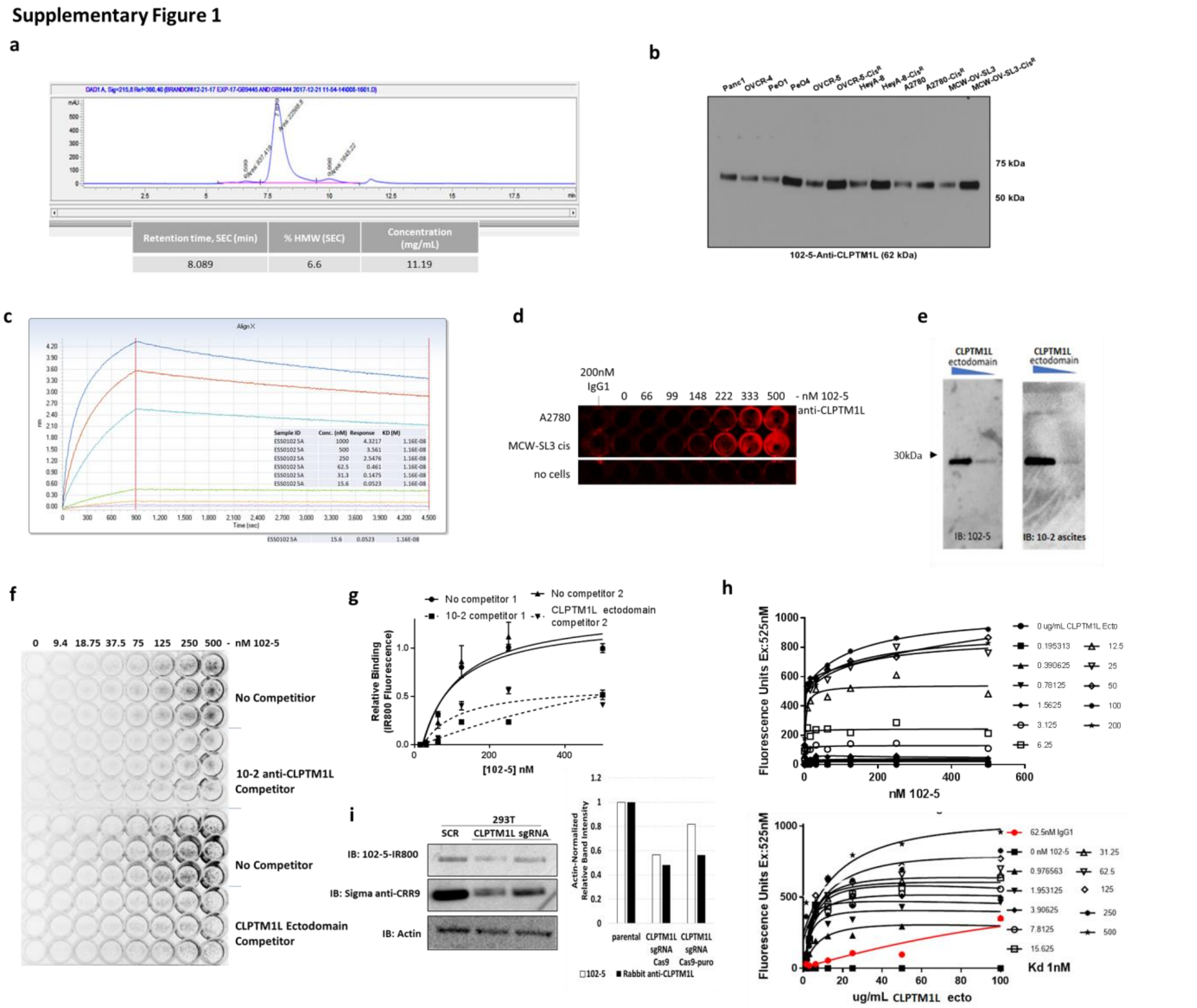

Supplementary Figure 1. a) Size exclusion chromatography of purified 102-5 anti-CLPTM1L in PBS demonstrating a single peak at the expected retention time consistent with highly pure IgG1. b) Full-length western blots using 102-5 anti-CRR9 on lysates from tumor cells. c) Biolayer interferometry affinity measurement of 102-5 anti-CLPTM1L to the CLPTM1L peptide epitope. d) On-cell western on tumor cell lines grown to confluence on 48 well plates and incubated with antibodies conjugated with IR-800 near infrared fluorophore (LiCor) on A2780 and MCW-SL3 human ovarian with 0-500 nM anti-CLPTM1L 102-5-IR-800 or 200 nM IgG1 control and e) Western blotting of purified recombinant CLPTM1L ectodomain using 102-5 anti-CLPTM1L. f) On-cell westerns with CLPTM1L-specific unlabeled

inhibitors demonstrating specific binding of 102-5 anti-CLPTM1L to endogenous CLPTM1L on live Panc1 human pancreatic tumor cells. g) Plotting of least squares nonlinear curve fits for on cell binding with and without CLPTM1L-specific competitors. h) ELISA assays demonstrating binding and affinity to purified CLPTM1L ectodomain (~1 nM Kd as analyzed by two-site specific binding least squares nonlinear regression using GraphPad Prism ver. 7.04. i) Western blotting analysis of HEK293T cells with scrambled or CLPTM1L CRISPR Cas9 heterozygous knockout using either 102-5 anti-CLPTM1L or commercial Rabbit-anti-CLPTM1L.

Supplementary Figure 2

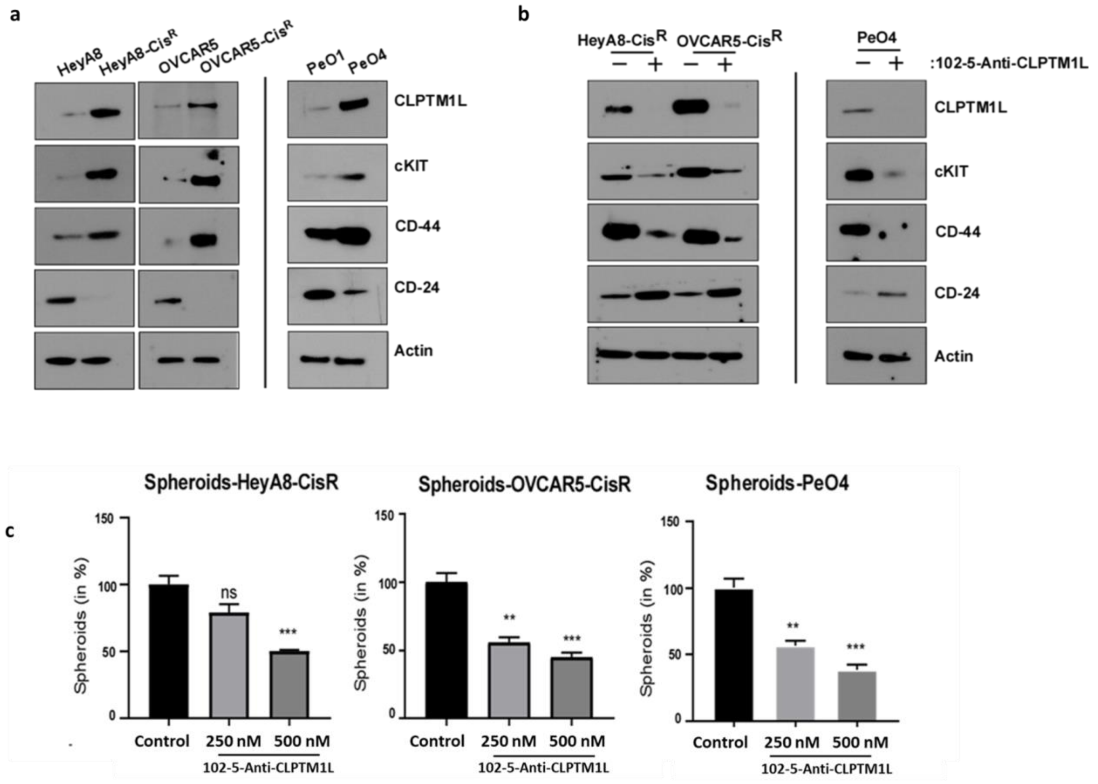

Supplementary Figure 2. a) Expression of CLPTM1L and stem markers in cisplatin-sensitive and resistant ovarian tumor cell lines. b) Expression of CLPTM1L and stem markers in cisplatin-sensitive and resistant ovarian tumor cell lines after treatment with 102-5-Anti-CLPTM1L antibody c) Dose-dependent inhibition of spheroid growth by 102-5 anti-CLPTM1L in cisplatin-resistant and ovarian tumor cell lines.

Supplementary Figure 3

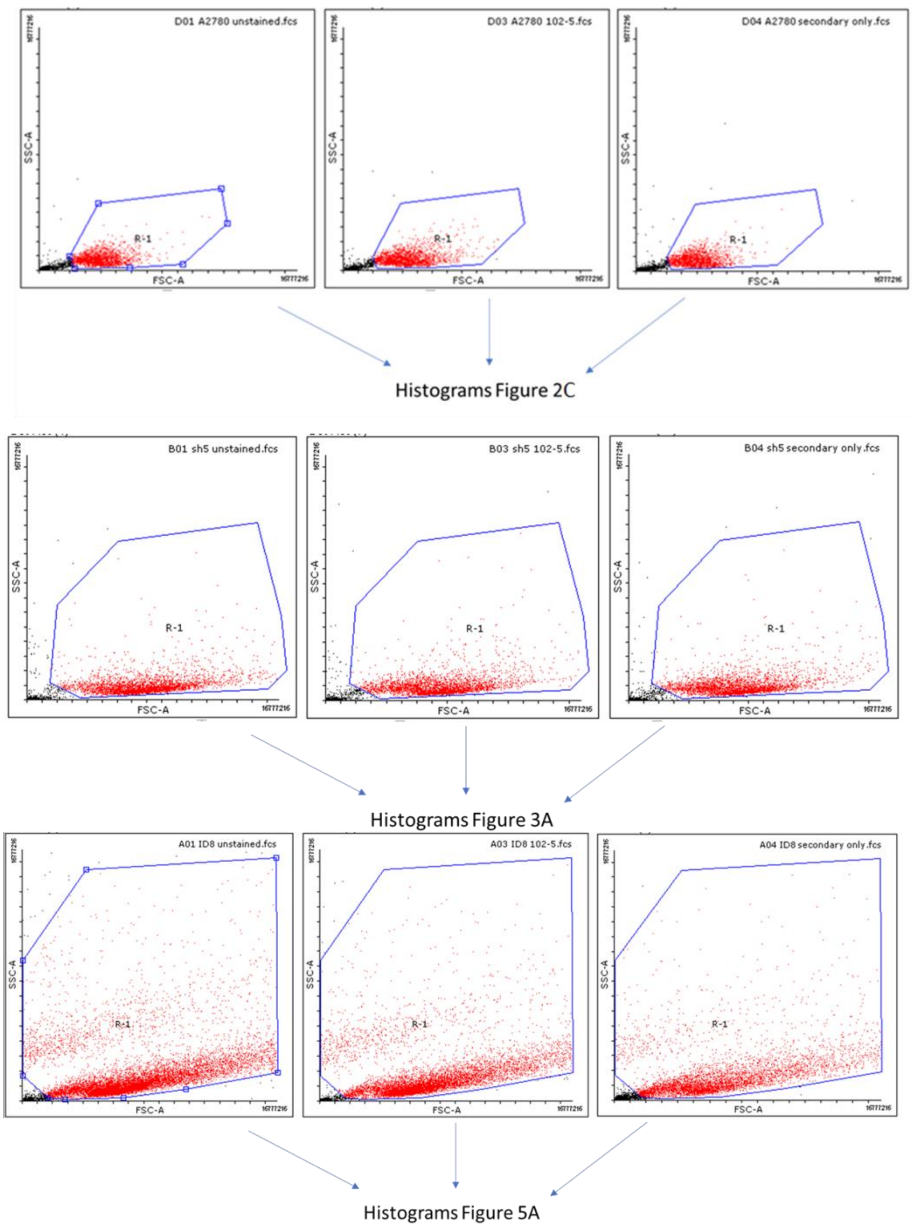

Supplementary Figure 3. Gating strategies for flow cytometry histograms

Supplementary Figure 4

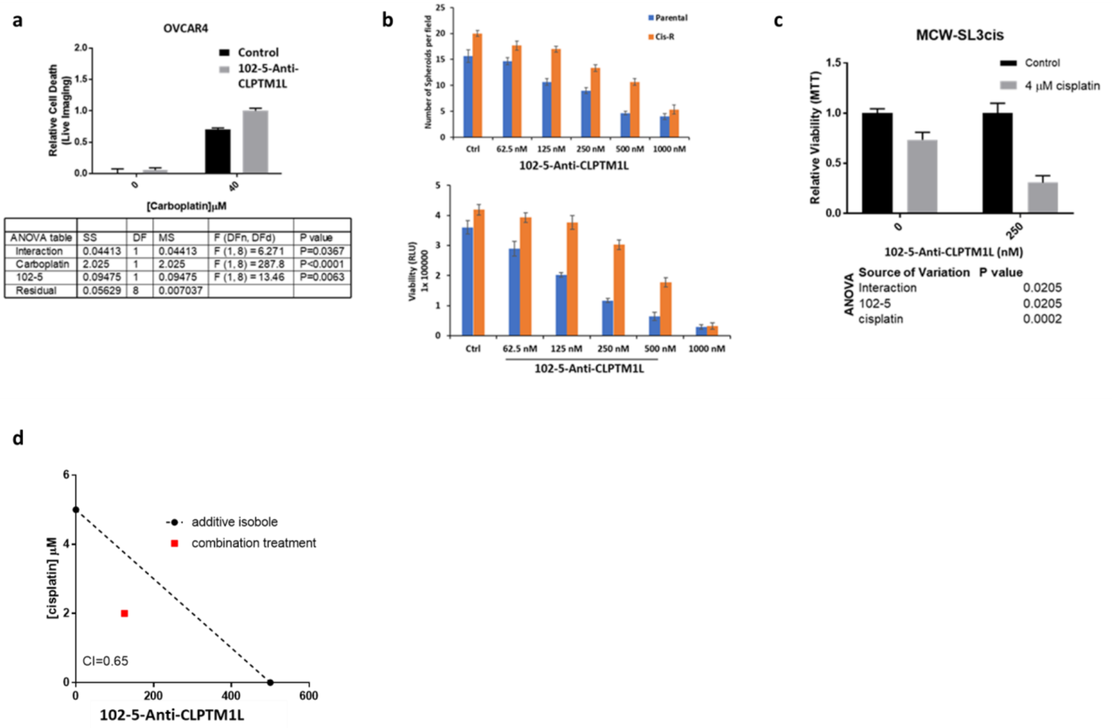

Supplementary Figure 4. Chemosensitization of ovarian serous adenocarcinoma and primary ovarian epithelial tumor cell lines by anti-CLPTM1L. a) Carboplatin cytotoxicity assay with and without 102-5 anti-CLPTM1L treatment. Four days carboplatin treatment after 24 hours treatment with 200nM 102-5. b) Anti-CLPTM1L 102-5 inhibition of MCW-OV-SL3 and MCW-OV-SL3-Cis^R spheroid growth. c) IC50 analysis for cisplatin in MCW-SL3 and MCW-SL3cis cells. d) Isobologram of anti-CLPTM1L 102-5 and cisplatin combination treatment of MCW-SL3cis spheroids at 50% inhibition of viability.

Supplementary Figure 5

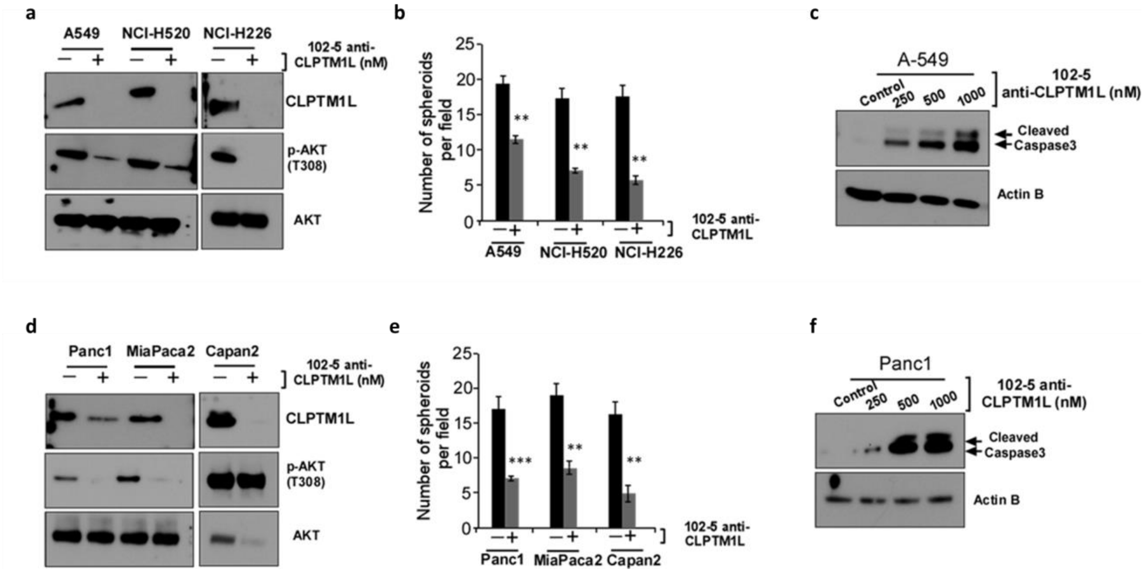

Supplementary Figure 5. Western blotting for CLPTM1L accumulation and Akt phosphorylation at T308 in lung (a) and pancreatic (d) tumor cell lines. Spheroid formation in lung (b) and pancreatic (e) tumor cell lines with and without 500 nM 102-5 anti-CLPTM1L. **p<0.01, ***p<0.001. Dose-dependent induction of Caspase-3 cleavage in lung (c) and pancreatic (f) tumor cell lines by 102-5 anti-CLPTM1L treatment.

Supplementary Figure 6

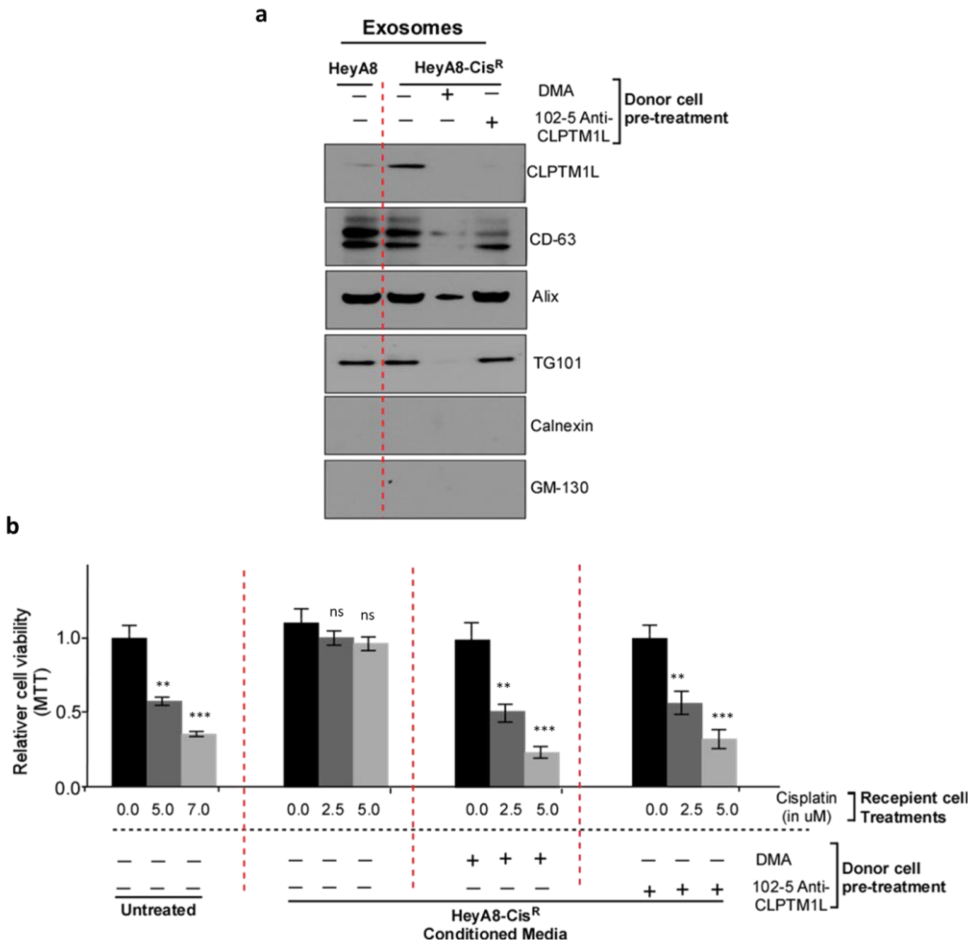

Supplementary Figure 6. a) HeyA8-cis^R cells were treated with or without DMA or 102-5 Anti-CLPTM1L and Western blotting from the exosomes isolated from the conditioned media using indicated antibodies. b) Cell viability was performed of HeyA8 cells treated with or without cisplatin that were untreated or pre-treated with culture supernatants of OVCAR5-Cis^R cells with DMA or 102-5 Anti-CLPTM1L (x axis).

Supplementary Figure 7

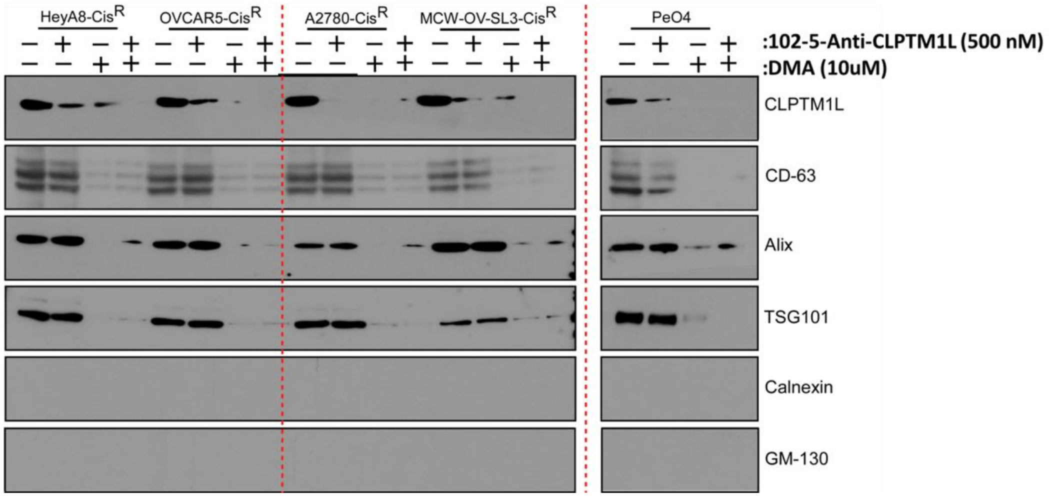

Supplementary Figure 7. Inhibition of CLPTM1L accumulation in culture supernatants of cisplatin-resistant ovarian tumor cell lines by the inhibitor of exosome production DMA.

Main Figure 1

E

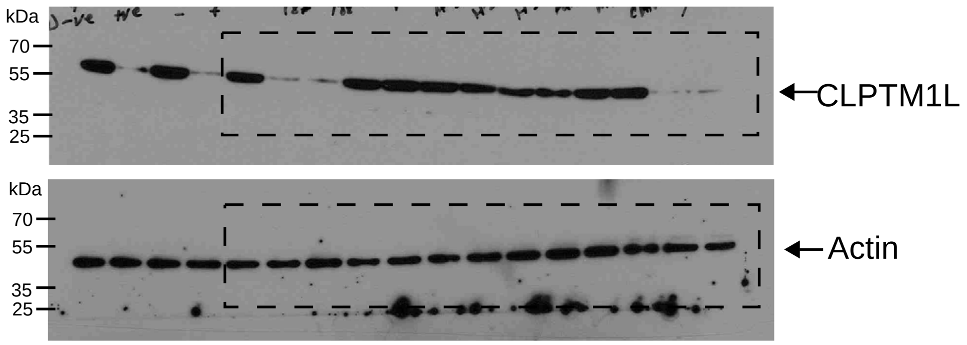

Main Figure 2

A

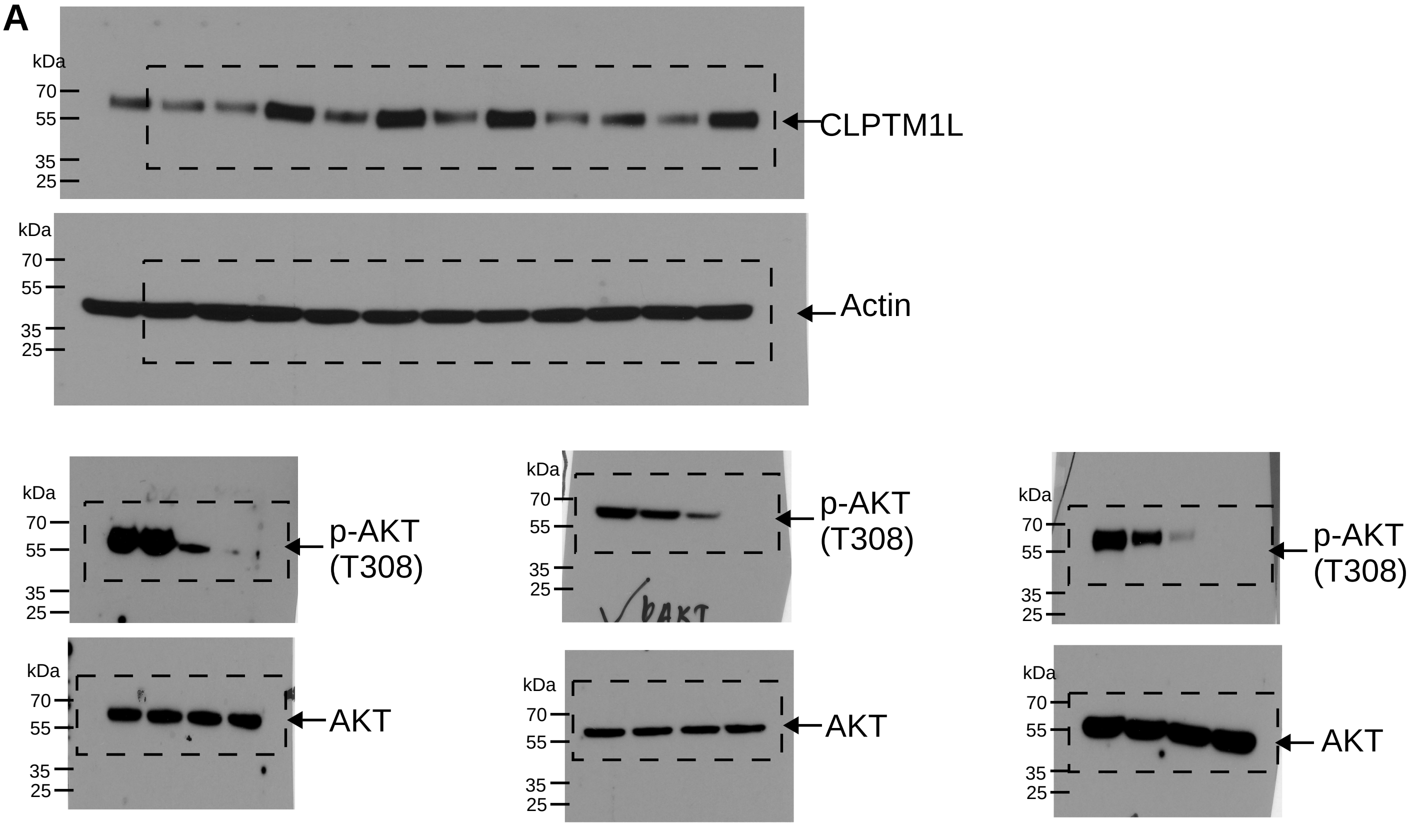

B

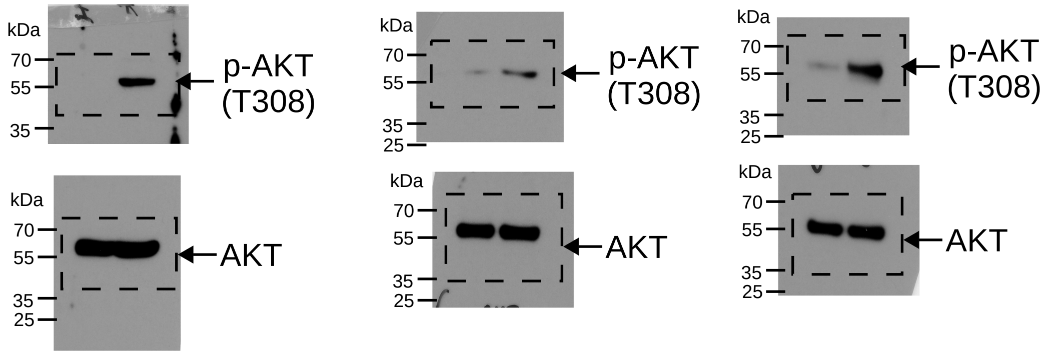

E

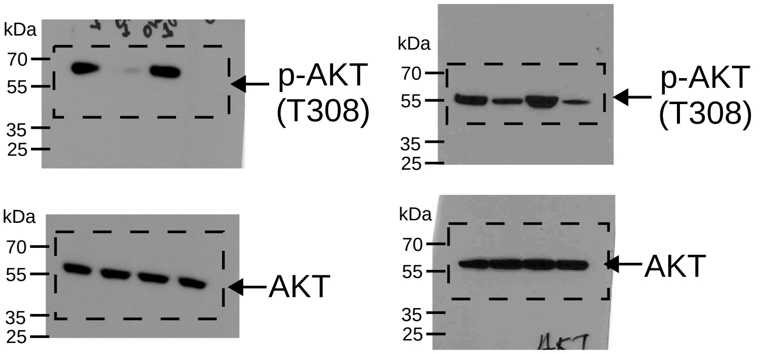

F

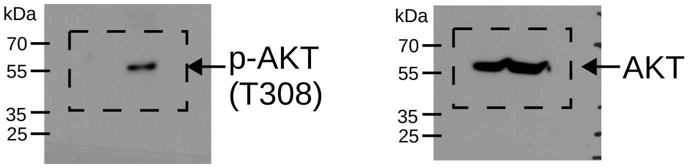

I

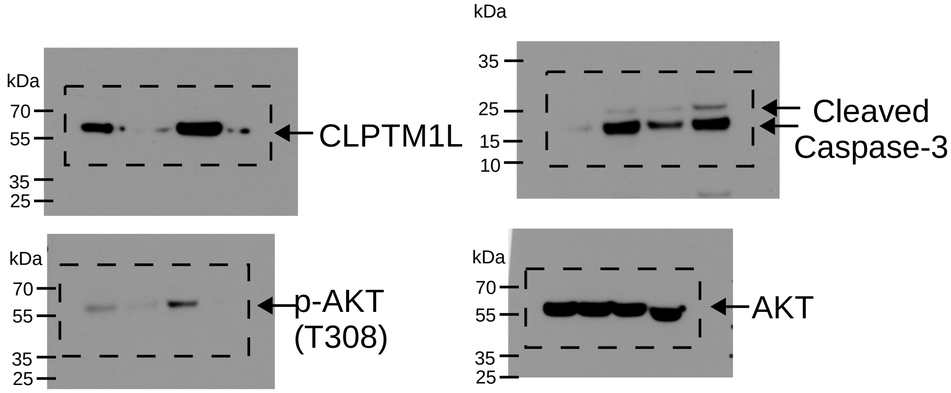

J

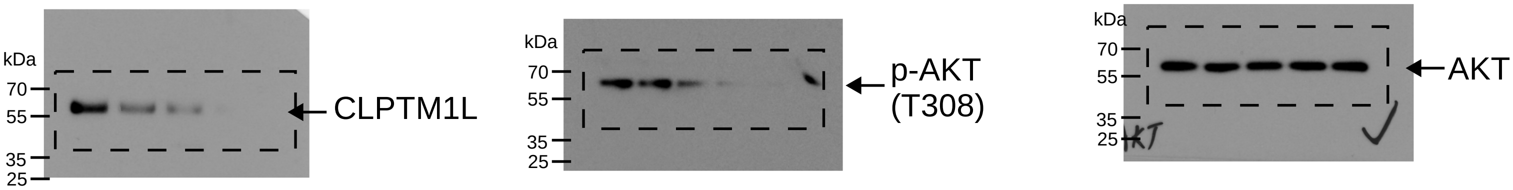

Main Figure 3

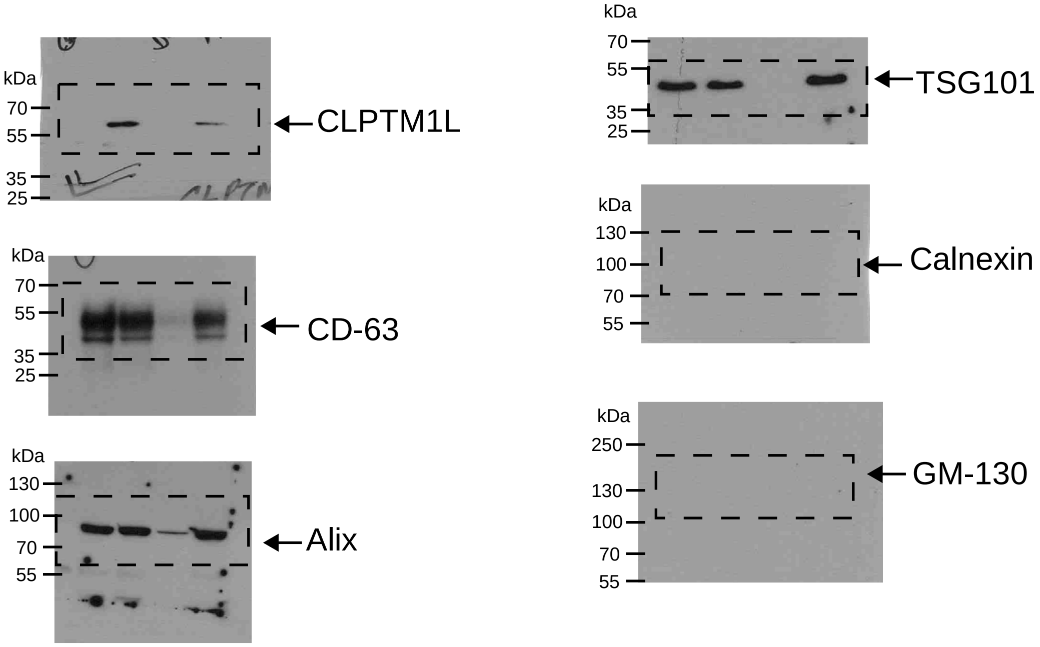

Main Figure 4

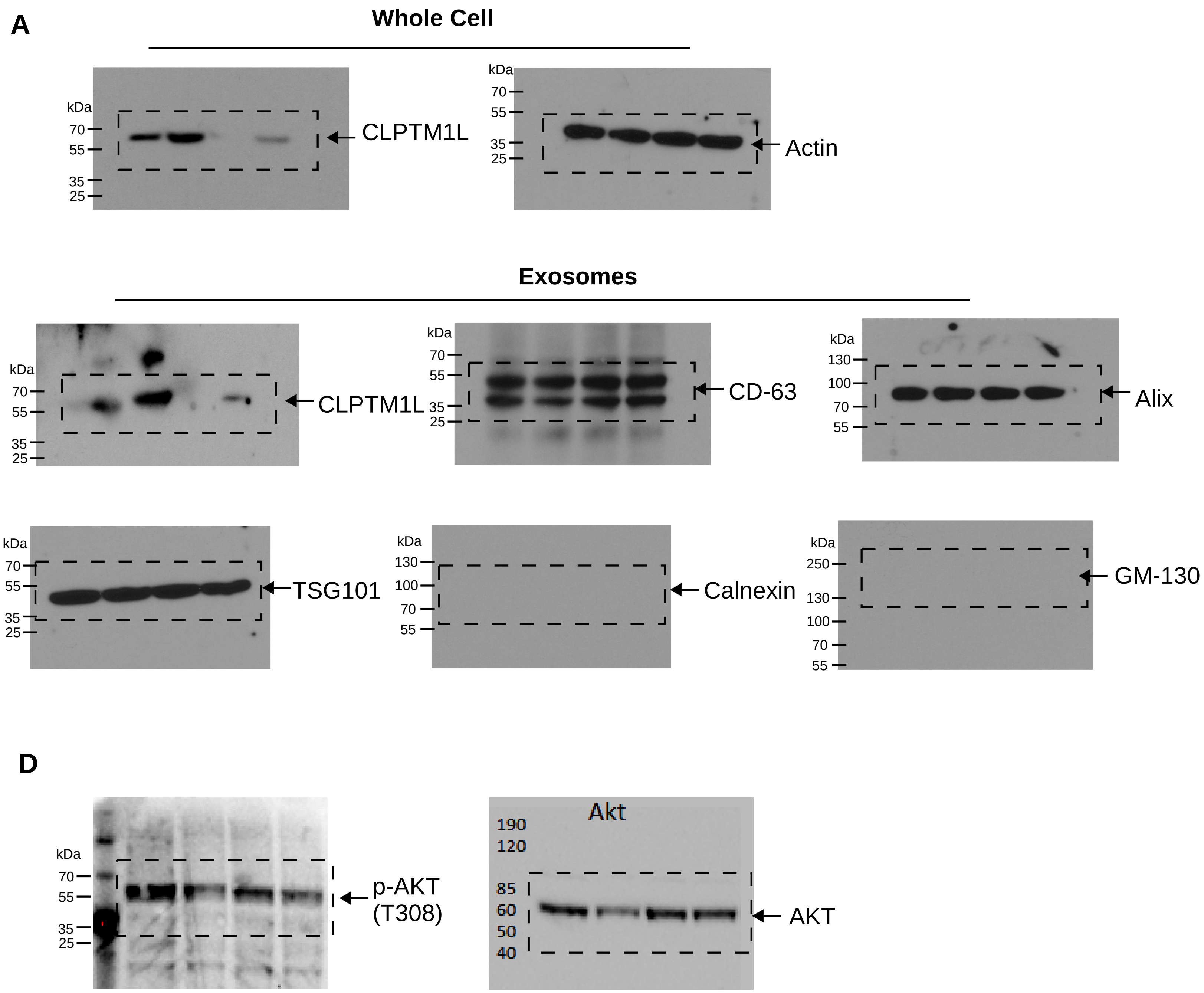

Supplementary Figure S1

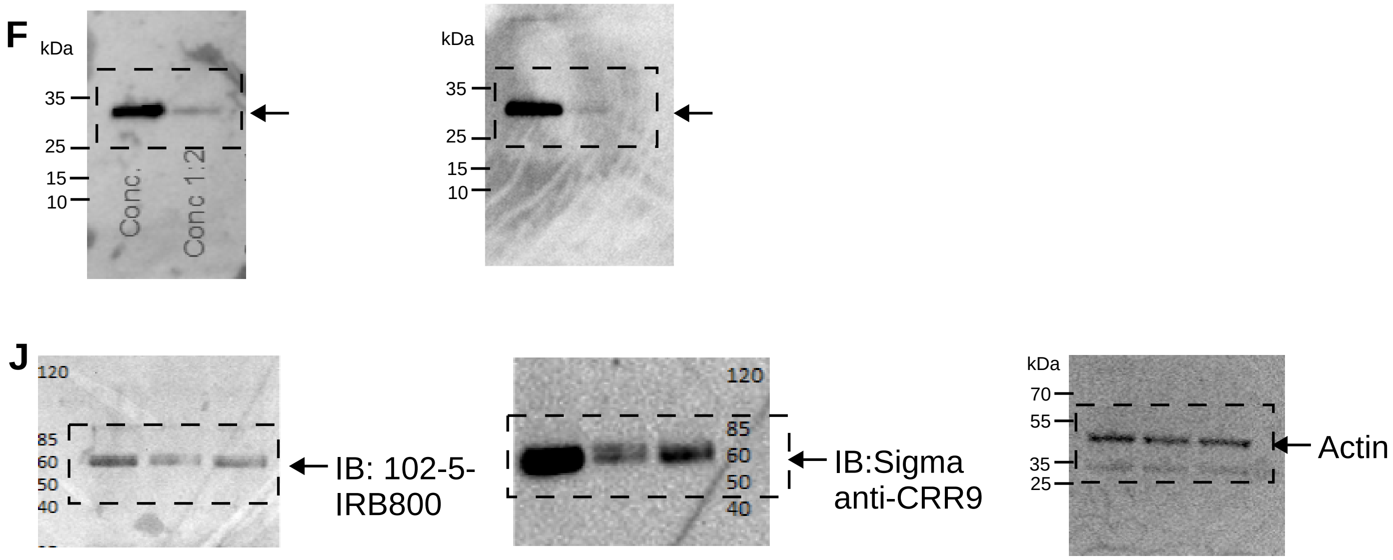

Supplementary Figure S2

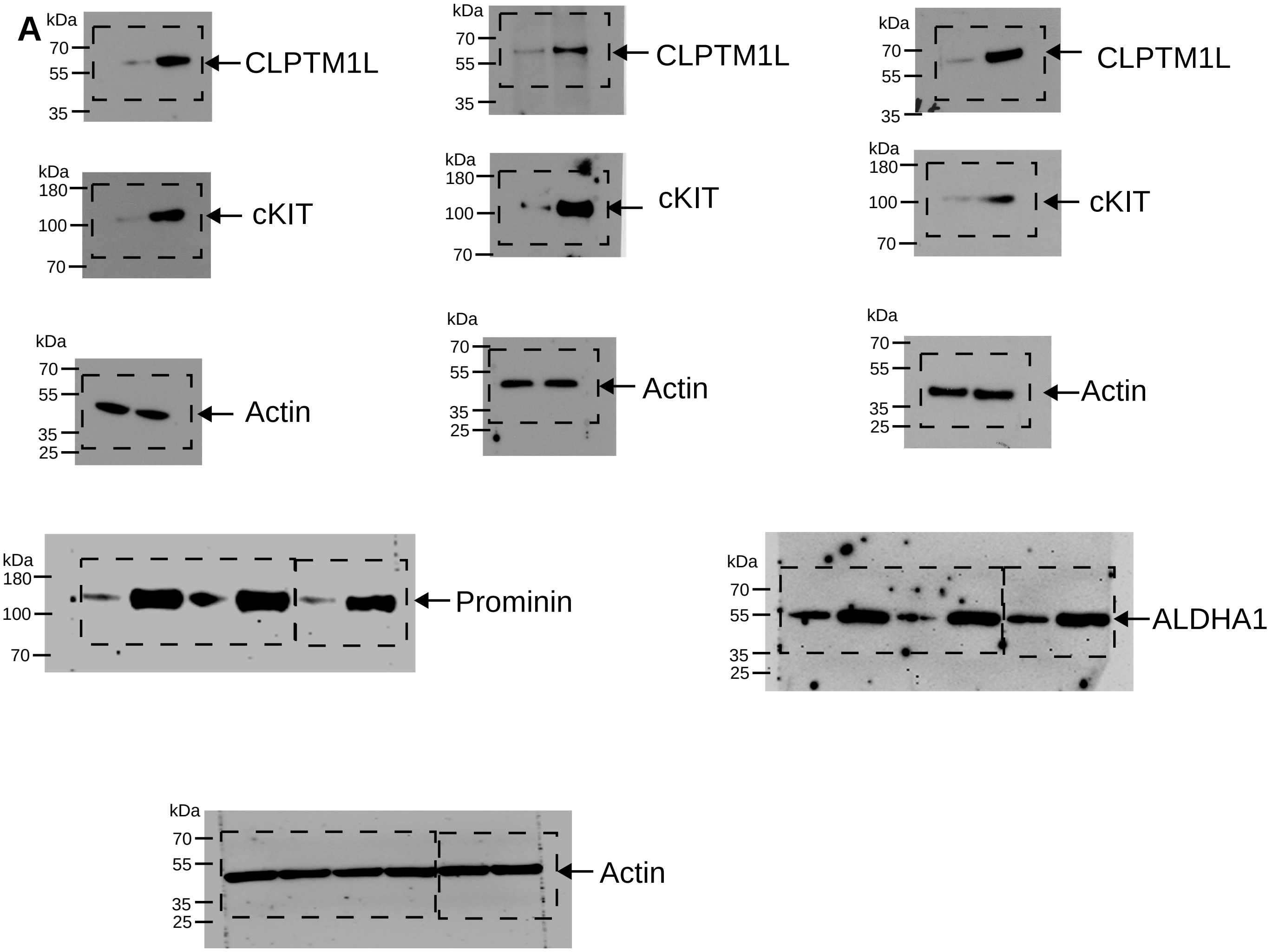

B

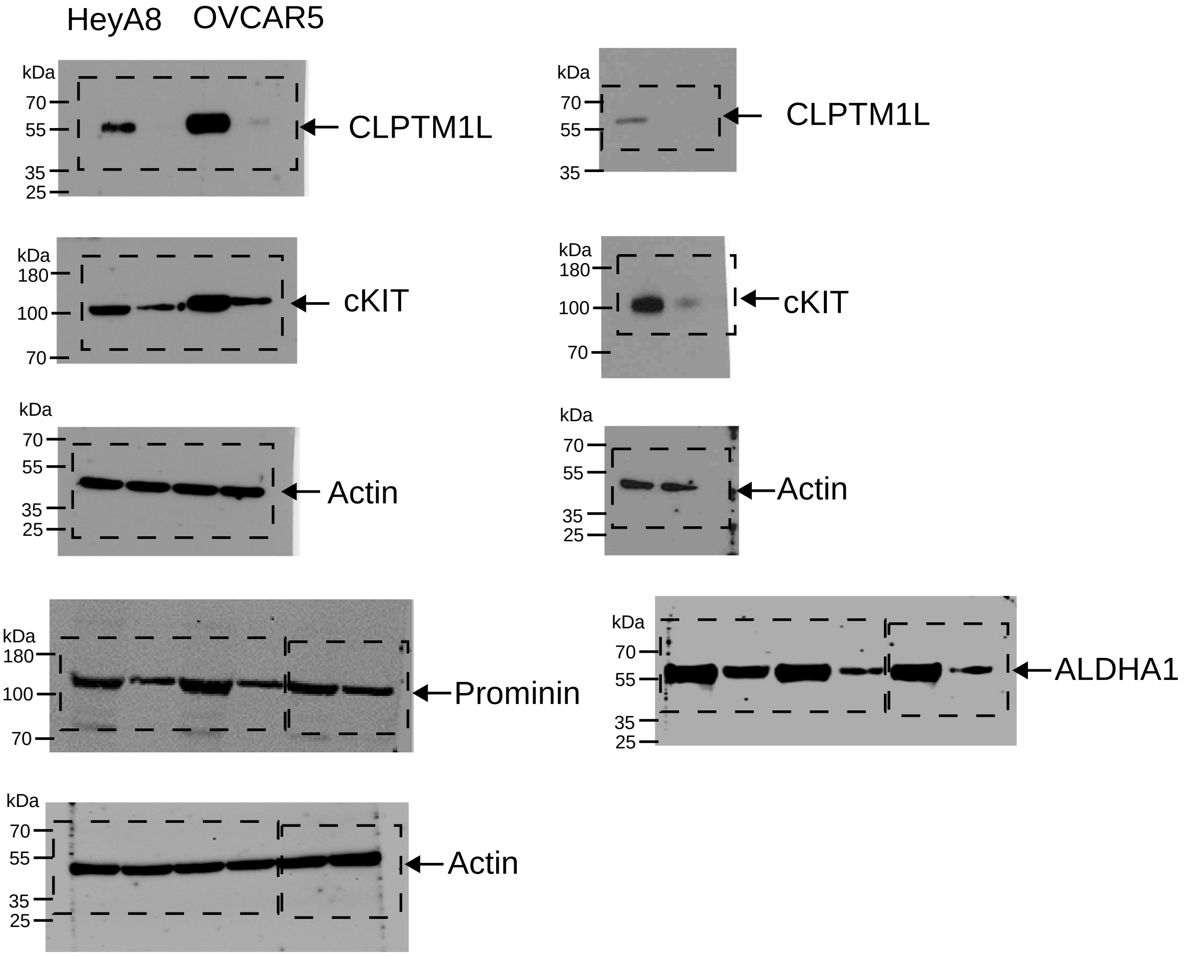

Supplementary Figure S5

A

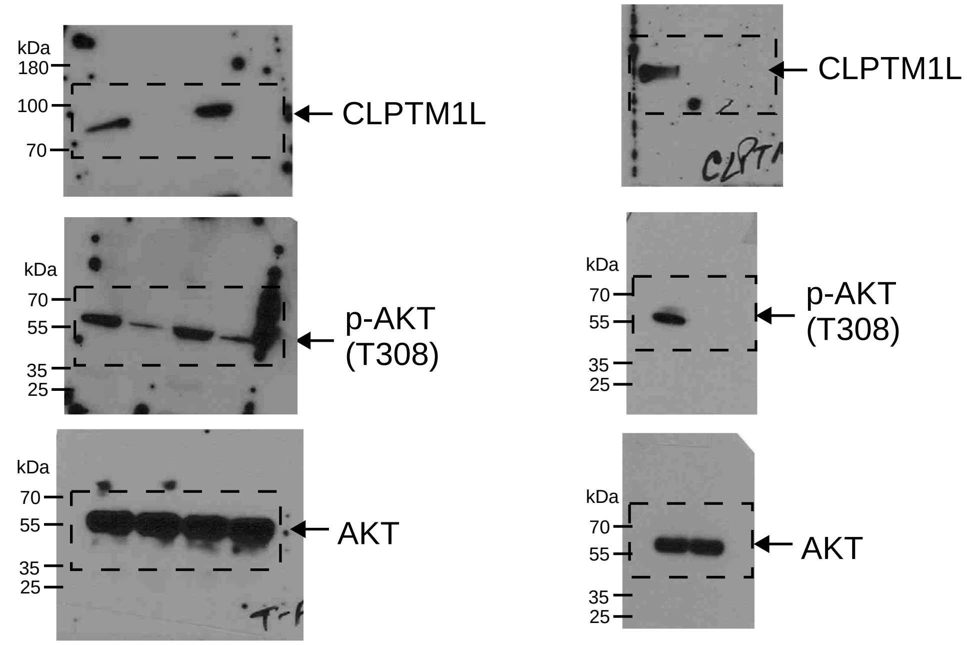

C

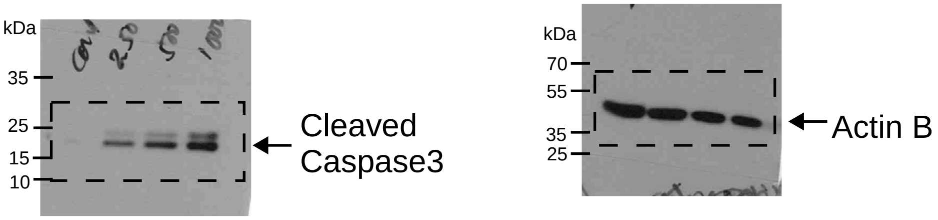

D

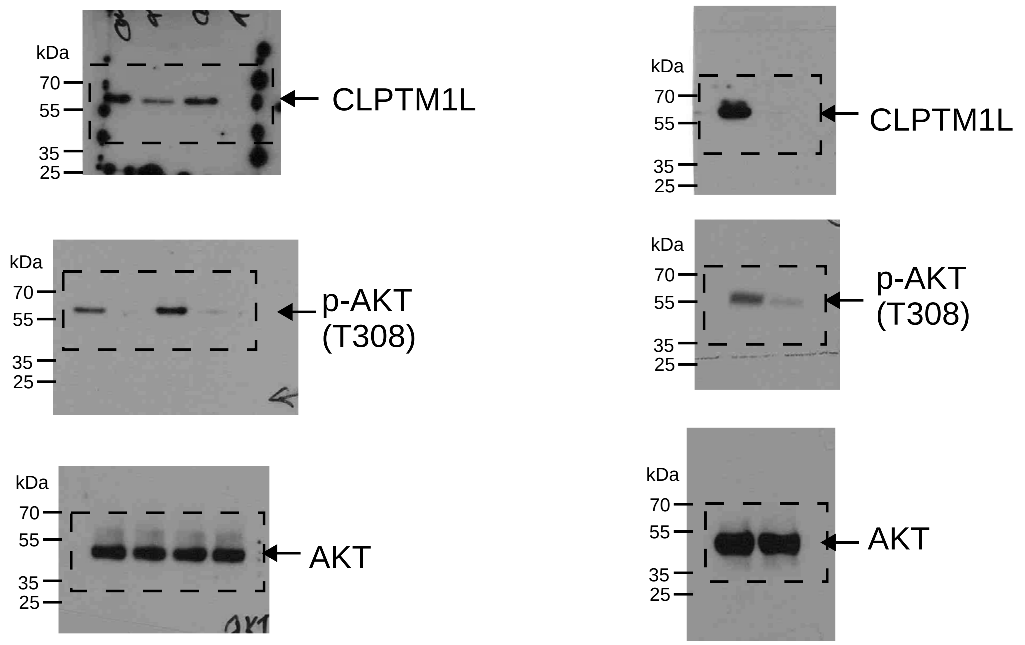

F

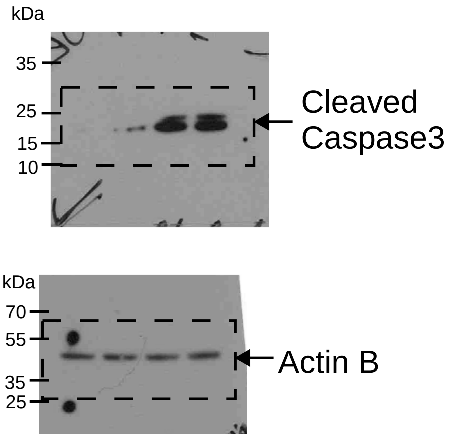

Supplementary Figure S6

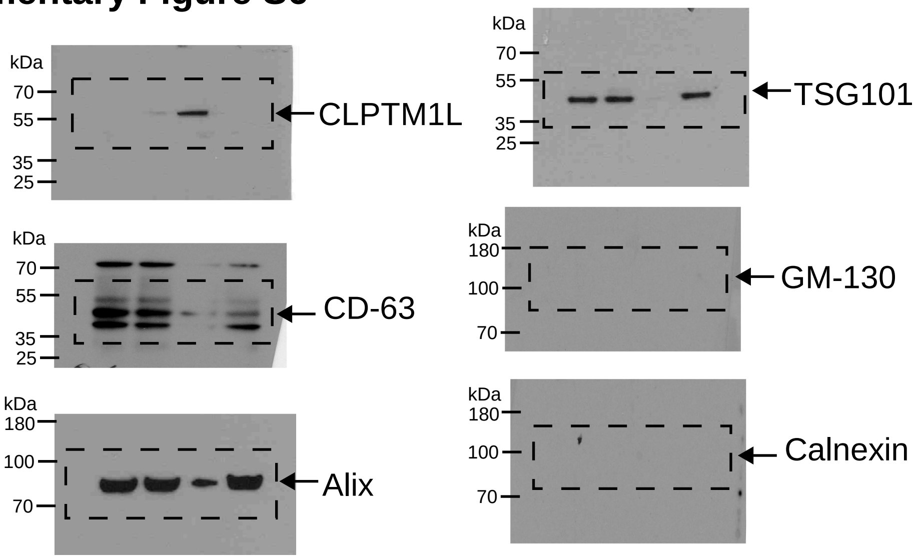

Supplementary Figure S7

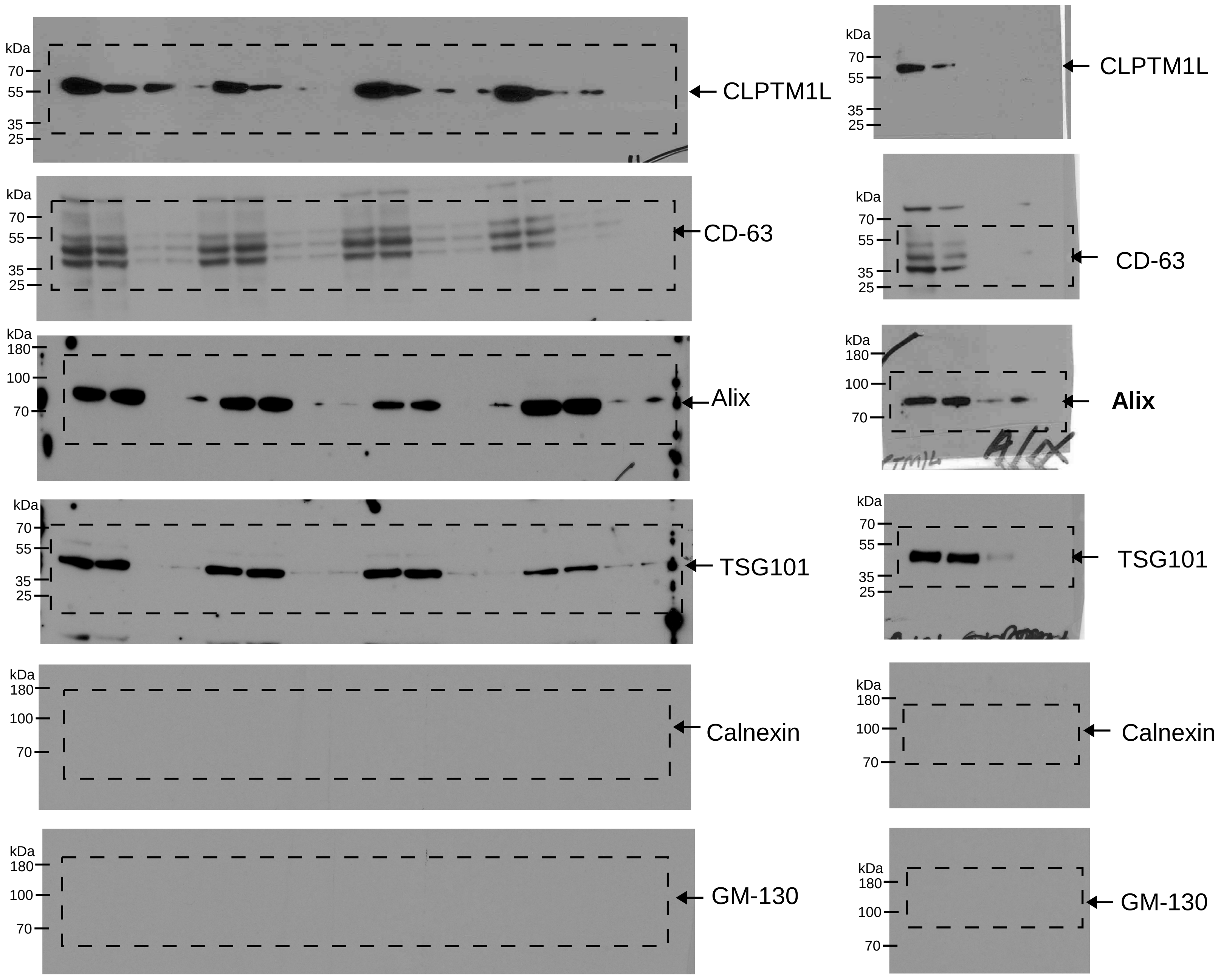

Supplement: Supplementary file 1 — Supplementary figures and legends [file 41698_2021_152_MOESM1_ESM.pdf]
